# Distinct transcription factor network dynamics underlie early and long-term adaptation to rifampicin in *Escherichia coli*

**DOI:** 10.1128/msystems.00808-26

**Published:** 2026-09-02

**Authors:** M. M. Azevedo, A. M. Arsh, R. Jagadeesan, Pooja Ravaria, Andre S. Ribeiro

**Affiliations:** 1 Laboratory of Biosystem Dynamics, Faculty of Medicine and Health Technology, Tampere University 7840 , Tampere, Finland; Rice University, Houston, Texas, USA

**Keywords:** genome-wide stress response, gene regulatory mechanisms, rifampicin, *E. coli*, *M. tuberculosis* orthologs

## Abstract

When subject to sublethal rifampicin stress, susceptible *Escherichia coli* cells quickly adapt to increase survival. We investigated whether the transcription factor network contributes to this adaptation. First, a responsive cohort of 709 genes diverged from the control, suggesting strong disturbance. While this was largely a direct effect of rifampicin, we show evidence that RNAP, σ^70^, σ^38^, Fnr, and (p)ppGpp were influential, suggesting early adaptations. We further show evidence that these responses can be promoter sequence dependent and are partially decoupled from changes in single-cell variability. Moreover, the responses of interacting genes were correlated, suggesting coordination. Later, the transcriptome partially realigned with the control, but 272 genes behaved consistently with long-term adaptation. Several exhibited correlated dynamics that can be explained by interactions, which form three independent, star-like modules of adaptive genes controlled by *gcvB*, *cdaR*, and *lldR*, respectively. Finally, we found that orthologous genes of the evolutionarily distant pathogen *Mycobacterium tuberculosis* have correlated response strengths to rifampicin, suggesting that our observations may be common across bacterial species. Overall, the results suggest that the initial effects of sublethal rifampicin concentrations on *E. coli*’s genome-wide transcriptional levels are dampened over time and then followed by a distinct state influenced by the gene network that may contribute to adaptation. These findings may assist in developing new strategies to disrupt bacterial adaptation to rifampicin.

In natural environments, exposure to sublethal antibiotic (AB) stress is a common phenomenon that enhances the emergence of AB resistance. We dissected the genome-wide transcriptional program of *Escherichia coli* responsible for its initial response and subsequent adaptation to rifampicin. We show that the transcription factor network plays a major role in this program and provide evidence that the transcriptome response patterns are conserved in the evolutionarily distant pathogen *Mycobacterium tuberculosis*. Our findings may assist in developing new strategies to disrupt bacterial adaptation to rifampicin.

## INTRODUCTION

In natural environments, bacteria are commonly exposed to sublethal antibiotic (AB) stresses. For example, this occurs when they are relatively distant from the AB source, where concentrations are lower than the minimum inhibitory concentration (MIC) (1). These conditions facilitate the emergence of AB resistance mechanisms (2–8).

Since most ABs are toxic, susceptible bacteria trigger adaptation processes even at sub-MIC concentrations, such as metabolic changes (9–14). For example, *Escherichia coli* tunes several cellular processes to increase survivability to sublethal stresses and to transient exposures to lethal concentrations (15, 16). These AB-response processes and mechanisms involve energy metabolism, drug efflux pumps (e.g., AcrAB-TolC), quorum sensing, (p)ppGpp signaling, and toxin-antitoxin systems (17–20), among others. The gene regulatory mechanisms triggering these changes are yet to be identified.

Rifampicin is an AB that binds to RNAP holoenzymes, before (when free-floating) or when the RNAP binds to the DNA and forms an open complex, but not after that (21, 22). Once bound, rifampicin prevents RNAP from escaping promoters (21, 23). This hampers transcription, which, due to quick mRNA degradation rates, leads to quick reductions in mRNA levels (24). These reductions should be followed by (much slower) reductions in the corresponding protein levels, provided that cells continue to divide. What occurs in the gene regulatory network (GRN) afterward is largely unknown, but it eventually leads to adaptations in iron acquisition, DNA repair, aerobic respiration, and carbon metabolism that reduce the killing potency of this AB (25).

We investigated which GRN elements drive the transcriptional program(s) adapting *E. coli* to sublethal levels of rifampicin. For that, we exposed cells to sufficient rifampicin to disturb the transcriptome, but below the MIC, and then performed two time-resolved RNA-seq to identify the short- and long-term responsive gene cohorts. We then identified which genes are more likely to be involved in the AB adaptation. Next, we studied which regulatory mechanisms drive early responsive and/or adaptive genes. Finally, we investigated whether orthologous genes are involved in the response of *Mycobacterium tuberculosis* to sublethal rifampicin stress.

## MATERIALS AND METHODS

### Bacterial strains

We used K-12 MG1655 as the wild-type (WT) *E. coli* to collect RNA-seq and other data, unless stated otherwise. In addition, a YFP fusion strain library (26) was used to measure several single-cell protein levels, including SpoT and RpoB. Meanwhile, an RL1314 strain with RpoC endogenously tagged with GFP (generously provided by Robert Landick, University of Wisconsin-Madison) was used to quantify RNAP levels. Furthermore, the activity of *fnr* was measured using a strain from a library of single-copy plasmids with the promoter of interest controlling GFP expression (27).

Finally, a library generously provided by the Elf lab (28, 29) was used to study the influence of promoter sequences. The strains (engineered from K-12 MG1655) have a chromosomally integrated, constitutive, synthetic promoter pFAB120 (35 bp long). It is followed by one LacI binding site (20–21 bp long), a double ribosome binding site, RBS (33 bp long), a mVenus coding sequence (717 bp), and a double terminator sequence (57 bp).

The LacI binding site differs between strains by one to a few nucleotides (Fig. 5A). These mutations alter the association-dissociation kinetics of the repressor, LacI (29). Since the repressor binding site is +3 to +21 bp downstream of the TSS, when LacI binds, it blocks RNAP escape from the TSS. This is also the step blocked by rifampicin (21, 22). Contrarily, LacI binding should not affect RNAP binding affinity to the promoter (29).

If these promoters were fully induced (500 µM IPTG or higher), the mutations in the LacI binding site would not be influential, since LacI will only rarely (or never) bind. Similarly, under full repression (0 µM IPTG), the mutations should also not be influential, since RNA production is blocked (unless the mutations sharply weakened LacI binding affinity). Thus, to study the effects of rifampicin on these promoters, we used half-inductions (250 µM).

Finally, we measured the protein levels in these cells, first at 50 min after adding both IPTG and rifampicin and then again 40 min later. The first 50 min should suffice for IPTG to enter cells and activate promoters (~10 min) (30), which will increase protein levels, given the fast rates of RNA production (~10 min^−1^) and protein production and maturation (mVenusNB, 4.7 min^−1^ at ~30°C [31]). The subsequent 40 min should suffice for protein levels to decrease, given rifampicin intake time (5 min) and mRNA degradation rate (~0.8 min^−1^) (29). For control, we performed the same measurements in the absence of rifampicin for each strain.

### Growth conditions and rifampicin

From glycerol stocks stored at −80°C, we streaked cells on lysogeny broth (LB) agar plates with ABs and incubated overnight at 37°C. Next, for all measurements, we independently selected three single colonies and inoculated them into fresh LB medium and incubated overnight at 37°C with appropriate ABs and aeration at 250 rpm. From each overnight culture, we diluted cells (1:1,000 in LB medium) and incubated them at 37°C with aeration. Finally, cells grew until reaching an optical density of ~0.3 at 600 nm (OD_600_, measured with a Biotek Synergy HTX Multi-Mode Reader), with rifampicin being added at that moment.

In the experiment in which IPTG was added, it was added at an OD_600_ of ~0.3. After 50 min, rifampicin was added. Measurements were conducted 40 min later.

Notably, rifampicin has been used to measure mRNA degradation rates (24, 32–40). This requires RNA production to be fully halted, which occurs under highly lethal rifampicin concentrations (50–500 µg/mL). Other studies (41) focused on rifampicin resistance, using random point mutations to hamper rifampicin’s binding affinity (21, 42). These past data are not used here to study signal propagation between genes.

### Minimum inhibitory concentration

We measured MIC as in reference 15. Overnight cultures were diluted 1 in 10,000 in fresh LB medium, grown with twofold dilutions of rifampicin, and incubated for 24 h at 37°C and 250 rpm shaking.

### RNA-seq

We performed RNA-seq at 80 and 160 min after adding rifampicin and in its absence for control (three biological replicates per condition). The data for 160 min have been used in reference 13, whereas the data for 80 min are first published here. Measurements and data analysis were performed using the methodology described in reference 13, which is also described below. Since some genes were removed from the analysis, cohort sizes can differ between 80 and 160 min.

#### Sample preparation

Cells in the mid-exponential growth phase (OD_600_ ~ 0.3) were subjected to rifampicin (2.5 ng/μL). After 80 and 160 min, we collected cells from each independent biological replicate. We also collected cells not subject to AB (control). After that, 5 mL of the culture was treated with a double volume (10 mL) of RNA protect bacteria reagent (Qiagen, Germany) for 5 min at room temperature to prevent RNA degradation. Next, cells were pelleted and frozen at −80°C. The next morning, total RNA was extracted using the RNeasy kit (Qiagen, Germany).

#### Sequencing

RNA was treated twice with DNase (Turbo DNA-free kit, Ambion, USA) and quantified using Qubit 2.0 Fluorometer RNA assay (Invitrogen, Carlsbad, CA, USA). The total RNA quality was determined by 1% agarose gel staining with SYBR Safe (Invitrogen, Carlsbad, CA, USA) using UV in a Chemidoc XRS imager (Bio-Rad, USA). RNA integrity was measured by Agilent 4200 TapeStation (Agilent Technologies, Palo Alto, CA, USA). Finally, RNA library preparations, sequencing, and quality control of sequenced data were performed by GENEWIZ, Inc. (Leipzig, Germany). Meanwhile, sequencing libraries were multiplexed and clustered on one lane of a Flowcell, which was loaded on an Illumina NovaSeq 6000 instrument. The samples were sequenced using a single-index 2 × 150 paired-end configuration. Image analysis and base calling were conducted using the NovaSeq Control Software v1.7 (Illumina NovaSeq). Raw sequence data (.bcl files) were converted to “fastq” files and de-multiplexed using Illumina bsl2fastq v.2.20. One mismatch was allowed for index sequence identification.

#### Data analysis pipeline

Step 1, RNA sequencing reads were trimmed to remove adapter sequences and nucleotides with poor quality using Trimmomatic v.0.39 (43); step 2, to generate BAM files, trimmed reads were mapped to the reference genome *E. coli* MG1655 (NC_000913.3) using Bowtie2 aligner v.2.3.5.1 (44); step 3, unique gene hit counts were calculated using “featureCounts” from the “Rsubread” R package (v.2.8.2) (45). Genes with less than five counts in more than three samples or whose mean counts were smaller than 10 were removed; Step 4: the DESeq2 R package (v.1.34.0) (46) was used to calculate log_2_ fold changes (LFC) of RNA read counts between conditions and the corresponding *P*-values, using Wald tests (function “nbinomWaldTest”). Since some genes were removed from the analysis at 80 and/or 160 min, gene cohort sizes can differ between the 80- and 160-min data sets. Finally, *P*-values were adjusted for multiple testing using the Benjamini–Hochberg false discovery rate correction.

### Flow cytometry

We used a NovoCyte Flow Cytometer and the Novo Express software (ACEA Biosciences Inc.). Cells were diluted (1:10,000) in 1 mL of PBS solution and vortexed for 10 s. We collected three biological replicates per condition, 50,000 cells per replicate. Flow rate was 12 µL/min.

To detect GFP, YFP, and mVenusNB, we used the FITC channel (-H parameter) with 488 nm excitation, 530/30 nm emission, and 14 μL/min sample flow rate, with a core diameter of 7.7 μm. PMT voltage was set to 600 for FITC, and sample flow rate was set to 14 μL/min, with a core diameter of 7.7 μm. PMT voltage was 584. To remove data from particles smaller than bacteria, the detection threshold was set to 5,000. We also collected the “pulse width” to use as a proxy for cell size (47, 48). No gating was applied. All events were collected by the Novo Express software from ACEA Biosciences Inc.

### Spectrophotometry

We monitored cell growth from optical density at 600 nm with a Biotek Synergy HTX Multi-Mode Reader.

### Cellular ATP

We grew QUEEN-2m cells, a kind gift from Hiromi Imamura of Yamaguchi University in Japan (49), in standard growth conditions, with and without rifampicin. We tracked ATP using a Biotek Synergy HTX Multi-Mode Reader, exciting the solution at 400 nm and recording the emission at 513 nm. Then, the solution was re-excited at 494 nm, and the emission was recorded at 513 nm. The ratio between the 513 nm emission intensities at the two excitation wavelengths, denoted “400ex/494ex,” was used as a proxy for cellular ATP levels (49).

### Statistical tests

To identify local peaks in the single-cell protein expression distributions, non-parametric kernel density estimates were generated using MATLAB’s ksdensity function with a Gaussian kernel. Local maxima were subsequently detected using the MATLAB function “findpeaks.” To reduce false-positive peaks, only peaks with a prominence greater than 5% of the maximum density value were considered.

For comparing distributions, *P*-values were obtained from two-sample *t*-tests to assess whether two distributions were from independent random samples from normal distributions with equal means and equal but unknown variances. For *P*-value <0.05, we rejected the null hypothesis that the two distributions have equal means.

To determine whether the slope of the linear fit differed significantly from zero, we calculated the *P*-value using the ordinary least-squares method.

When the results were uncertain, i.e., when they could be affected by the removal of a small number of data points, we also calculated the Pearson correlation coefficient to determine whether the two variables were linearly correlated or not.

Meanwhile, we considered the results “uncertain” when the *P*-value of the ordinary least-squares linear fit was close to 0.05, since in such cases, the conclusion could change upon removal of a small number of data points.

Finally, we used null models to test if correlations between LFC values of genes interacting via TFs are due to the interactions. Null models were obtained by shuffling the LFCs of output genes, while maintaining the LFCs of input genes. If the correlation in random models equaled that of the real model, we would conclude that the correlation is not due to the TF interactions.

### Network topology

To analyze network topologies, we studied the number, directionality, and regulatory roles of inputs and outputs of each gene (including self-inputs). sRNA interactions were also included (~350 involving 40 sRNAs, representing ~5% of all connections). Overall, they did not show distinct behavior or affect qualitative conclusions, except for the contribution of gcvB to the network among adaptive genes.

We obtained clustering coefficients (number of connections between a node’s nearest neighbors over the number of possible connections), outdegree centrality (standard deviation of outdegrees over mean network outdegree), and shortest path length (number of connections of the shortest directed path between two genes). Finally, we identified the top-three share of outdegrees (sum of the outdegrees of the three nodes with higher outdegrees divided by the sum of the outdegrees of all nodes).

## RESULTS

### Expected timing of the main GRN-driven events triggered by rifampicin

**Fig 1:**
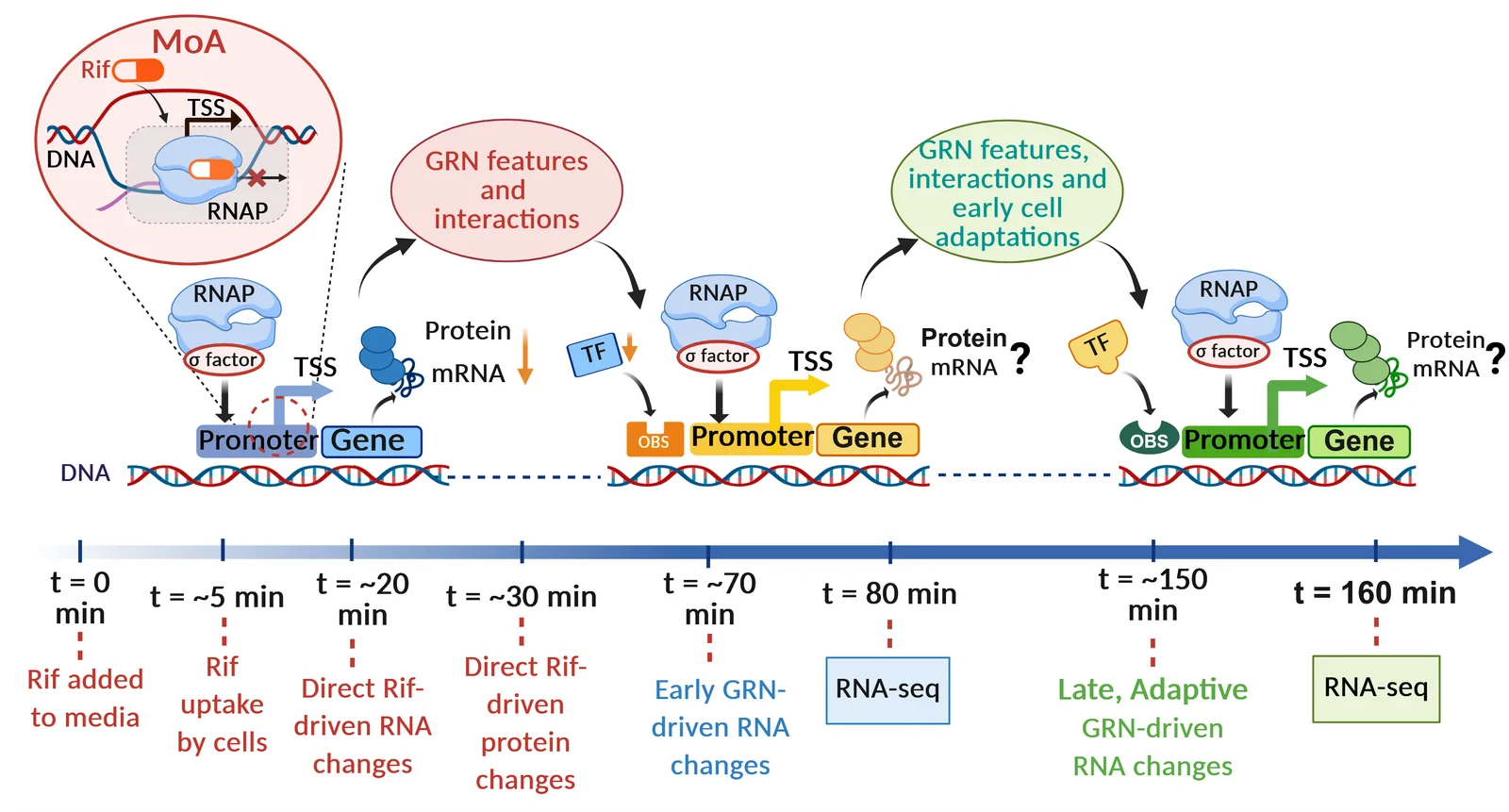
Illustration of the expected timeline of early GRN-driven stress-related and late adaptation-related events on the transcriptome of *E. coli*, triggered by rifampicin. Image adapted from reference 51. We predict three main phases. First, there will be quick genome-wide reductions in RNA and corresponding transcription factor levels, directly caused by rifampicin. Second, there will be an “early” GRN-driven phase of changes, already influenced by GRN features and interactions, but still largely a consequence of the initial rifampicin-based perturbations. Finally, we expect late changes, mostly driven by the GRN and more likely to lead to beneficial AB adaptations. TSS, transcription start site; MoA, mode of action; and Rif, rifampicin.

Figure 1 illustrates the main events expected to be triggered by sublethal rifampicin stress, given this AB mode of action (21). First, once added, rifampicin should enter cells quickly (less than 5 min [50]), bind to RNAPs, and block their escape from promoters, decreasing both RNA production rates and free-floating RNAP concentrations (51).

In *E. coli*, ~50% of all genes should be active during exponential growth in standard conditions (26). Their RNA numbers should quickly decrease under rifampicin. Since cells have 10 or fewer RNAs per active gene in this growth phase (26), and mRNA half-lives are ~4–7 min (24, 26), several RNAs can become undetectable within ~15 min. Subsequently, corresponding protein levels will drop by degradation and dilution by cell division (52). In the exponential growth phase, protein numbers could change significantly within ~30–40 min after adding rifampicin (26, 31, 53, 54). Here, we refer to these changes of the transcriptome and proteome (largely reductions due to transcriptional locking) as the initial changes directly caused by rifampicin.

It should follow a phase of global changes partially driven by TF and other gene-gene interactions (13, 51). Interestingly, in *E. coli*, ~52% of all TF interactions are activators, while ~47% are repressors (51, 55). The reduced activity of activators due to rifampicin should further reduce the activity of their target genes (in addition to reductions caused directly by rifampicin). Conversely, the reduced activity of repressors should have the opposite effect on their target genes, i.e., should enhance their activity. Thus, during the “early GRN-driven phase,” the TF network (TFN) is expected to, at least partially, reverse the initial RNA reductions of many genes under negative TF regulations. Given the above-mentioned time lengths for mRNA and protein numbers to change, to study this phase, we performed an RNA-seq at 80 min after adding rifampicin. Since most of these changes are likely a direct consequence of the initial effects of rifampicin, many are not likely to contribute to the adaptation to rifampicin.

Finally, we expected “late” changes in RNA and protein levels. These are the most likely to drive the adaptation processes, such as the upregulation of efflux pumps (56, 57). Their mechanical triggers are yet to be identified. We hypothesized that the TFN plays a major role.

To study this phase, we performed a second RNA-seq, 160 min after adding rifampicin (Fig. 1). We expect the genes with activities different from those of the control at this stage are the most likely to be “adaptation-contributing” (adaptive) genes. To decrease false positives, we identified as “adaptive” only those genes that not only differ from the control at 160 min but also show a shift in expression pattern between 80 and 160 min. We interpreted this shift as evidence of a time-dependent behavioral reconfiguration, consistent with the existence of a transcriptional program responsible for enhancing survivability under rifampicin.

Finally, to reduce the potential influence of growth phase differences between time points, each gene’s response strength to rifampicin is the one relative to the same gene expression in the control condition at the same time point. Furthermore, to facilitate the study of the effects of reduced RNA production rates, we applied low levels of rifampicin that did not reduce cell growth, so as not to cause additional interference in the RNA and protein levels arising from changes in cell functioning.

### The sublethal rifampicin stress affected RNAP but not ATP or cell growth and size

**Fig 2:**
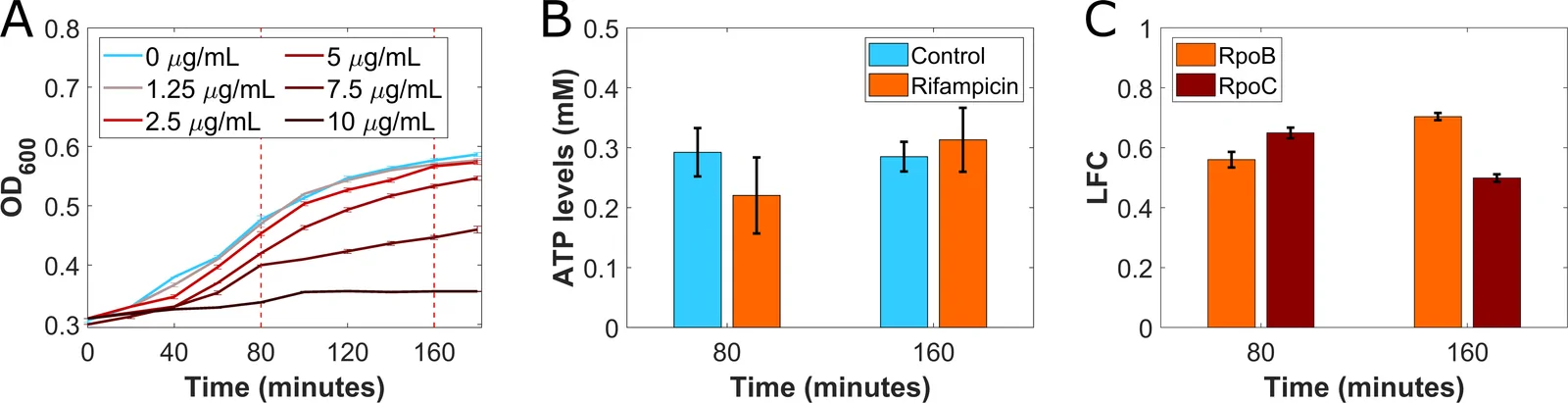
Cell growth, ATP, and RNAP under sublethal rifampicin levels. (**A**) Cell growth (optical density OD_600_) at various rifampicin concentrations. (**B**) ATP under 2.5 µg/mL of rifampicin at 80 and 160 min obtained by spectrophotometry. (**C**) Changes in the average single-cell expression levels of two subunits of RNAP (RpoB-YFP and RpoC-GFP, respectively) under 2.5 µg/mL of rifampicin, measured by flow cytometry. LFC stands for log_2_ fold change relative to the control condition. In all graphs, the error bars represent the standard error of the mean from three biological replicates.

To measure how weak rifampicin stress propagates in the GRN, we opted to minimize cellular damage. For this, we applied the maximum rifampicin concentration that did not affect the growth rate or morphology of the reference strain K-12 MG1655 (see “Bacterial strains”). We found it to be 2.5 µg/mL (Fig. 2A; File S1; see “Growth conditions and rifampicin”), in agreement with reference 57. For reference, the MIC (see “Minimum inhibitory concentration”) equaled 7.5 µg/mL (3× higher), similar to past reports (58).

Importantly, if the rifampicin concentration applied (2.5 µg/mL) does not decrease cell growth rates (Fig. 2A), reduced transcription should lead to decreased protein levels, since weaker production is combined with unchanged dilution rates. In addition, we considered the small difference between 2.5 µg/mL and control conditions at 40 min, because it was within the measurement variability of the instrument (±0.02), was not present at any other time point, and was not supported in the intermediate condition (1.25 µg/mL), we do not find it to be biologically significant.

We next tracked ATP (see “Spectrophotometry”” and Cellular ATP”; File S1), since it affects energy-consuming processes, such as DNA supercoiling regulation, which can hamper transcription and DNA replication (59). However, ATP levels did not change significantly (Fig. 2B). Similarly, the cell size, measured by the flow-cytometry parameter “pulse width” as a proxy (48) (see Flow cytometry), only increased by 4% relative to the control in 120 min (*P*-value of a two-sample *t* test <0.05 [see “Statistical tests”]).

Moreover, sublethal levels of rifampicin have been reported to transiently increase RNAP β and β′ subunits in *E. coli* (60–62) and *M. tuberculosis* (63). To test this, we measured RpoC:GFP in RL1314 cells under rifampicin (see “Bacterial strains”; see File S2 at https://doi.org/10.5281/zenodo.21899680). It was higher than the control at 80 and 160 min (Fig. 2C). We validated using a strain carrying RpoB-YFP (Fig. 2C) (see “Bacterial strains” and File S2). Again, RpoB was higher. This was also supported by the RNA-seq data (File S3). These increases should counteract the effects of rifampicin globally, not only by increasing free-floating RNAP levels but also by increasing the frequency of RNAP collisions in the DNA, which should free rifampicin-trapped RNAPs at promoter regions. This increase could partially explain many early increases in transcript levels reported below.

**Fig 3:**
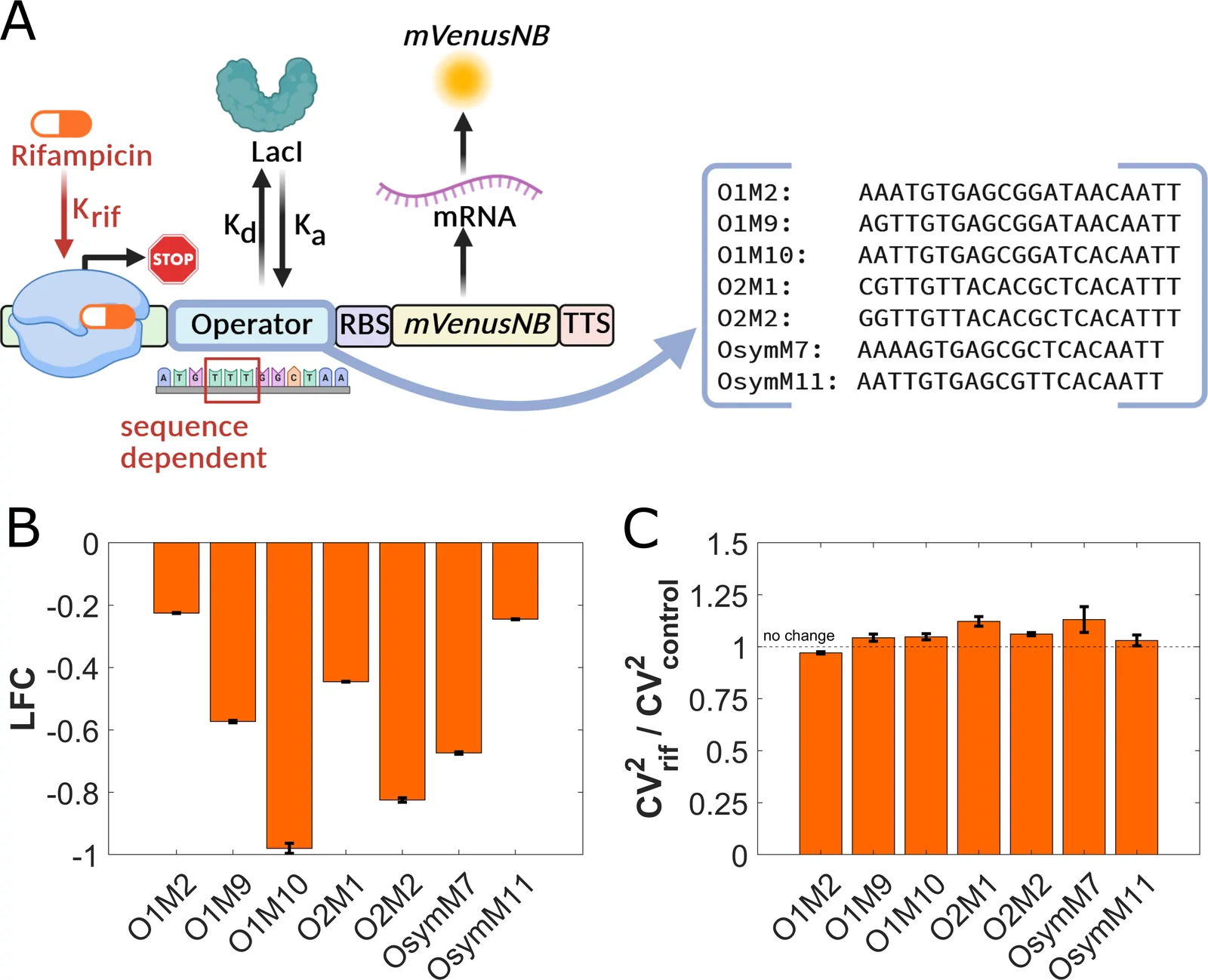
Mutations in the LacI operator region affect rifampicin effects. (**A**) Illustration of a promoter with an RNAP binding region (core promoter) and a binding site (operator) for the repressor LacI. The binding site sequence differs between strains, influencing the binding (*k*_*a*_) and unbinding (*k*_*d*_) rates of LacI (which may affect *k*_rif_, the binding affinity of rifampicin). Also shown are the coding regions, mRNA, and fluorescent mVenusNB proteins. RBS, ribosome binding site; TTS, transcription termination site. (**B**) Changes in mVenusNB protein expression levels relative to the control, 40 min after adding rifampicin. (**C**) Corresponding single-cell variability in mVenusNB protein levels relative to the control, estimated by the squared coefficient of variation (CV^2^). Error bars are the standard error of the mean from three biological replicates. LFC stands for log_2_ fold change relative to the control condition.

Meanwhile, past reports have suggested that, in certain conditions, rifampicin can have heterogeneous effects on cell populations, particularly at low concentrations (64). To test this, we measured single-cell distributions of expression levels of several native genes. From Fig. S1, there is single-cell variability in expression levels, but we failed to find bimodality. To further verify, we also studied chromosome-integrated synthetic promoters (pFAB120 with LacI binding sites) (Fig. 3). Again, we did not find significant bimodality (Fig. S2). We conclude that the measurements reported below, including RNA-seq, were obtained from cell populations affected relatively homogeneously by rifampicin.

We also searched for multiple local peaks in each of the single-cell distributions of gene expression above (see “Statistical tests”). We failed to find multiple peaks in any distribution at either 80 or 160 min. We conclude that the concentration of rifampicin applied did not affect only subpopulations of cells.

Finally, we performed a differential expression analysis between stationary-phase RNA-seq data (raw data from NCBI GEO with accession code GSE188752) against rifampicin-treated cells (160 min). We identified 1,186 genes significantly differentially expressed between conditions (Fig. S3, classified as differentially expressed if |LFC| > 1 and adjusted *P*-value < 0.05). This suggests that the transcriptome at 160 min differs widely from the transcriptome in the stationary growth phase.

We further considered the small difference in OD between 2.5 µg/mL and control conditions at 40 min, because it was within the measurement variability of the instrument, was not present in any other time point, and was not supported at 1.25 µg/mL, we do not find it to be biologically significant.

### Temporal profiling of direct and indirect responses to sublethal levels of rifampicin

As described above and illustrated in Fig. 1, we expect two major sources of changes in RNA levels. The first are the “direct” changes caused by rifampicin blocking transcription (in our conditions, cell growth is not affected and, thus, both mRNA and sRNA levels should change). Meanwhile, the GRN should cause “indirect” changes due to, e.g., changes in TF levels initially caused by rifampicin. Figure S4 shows the genome-wide distributions of LFCs at 15 (data from reference 40), 80, and 160 min after adding rifampicin, as measured by RNA-seq. Globally, the strongest changes occur during the first 15 min.

To dissect the contributions of direct and indirect sources in the overall changes in RNA levels over time, we considered the expected difference in the timings of both changes. Specifically, since the intake time of rifampicin is under 10 min and mRNA degradation rates are significant (29), the direct effects of rifampicin should be influential largely within the first ~20 min. These effects have been previously studied (see, e.g., reference 65). Subsequent changes should be largely due to indirect causes, such as changes in TF numbers. These have not yet been studied in detail and are our focus from here onward.

To provide support for the expected fact that first changes are directly due to rifampicin, whereas latter changes are largely more influenced by the GRN, we searched for correlations between changes in RNA levels (LFCs) of interacting genes (RegulonDB v. 13.0 [55]) at 15 (“immediate” changes), 80 (“early” changes), and 160 (“late” changes) min after rifampicin addition. Moreover, we compared the results with a null model by randomizing which genes are outputs of the interactions and, from 1,000 runs, selecting the one with the highest correlation. We use this null model to estimate the correlation resulting from the direct effects of rifampicin on all genes.

Results in Fig. S5 show that the smallest correlation between the LFCs of input-output genes occurs at 15 min (albeit it is higher than the null model). This correlation then increases significantly at 80 and 160 min, suggesting that the influence of gene-gene interactions on the state of the GRN increases over time.

To observe if this phenomenon propagates to protein expression levels, we tracked genes using the YFP strain library (26) using spectrophotometry under rifampicin and control conditions and then estimated relative values and calculated differences between each time point and the previous one to estimate changes over time. From Fig. S6, strong differences relative to the control occur ~40–80 min after adding rifampicin.

From here onward, we focused on the changes influenced by the GRN by studying the transcriptome at 80 and 160 min after rifampicin addition.

### Nucleotide changes in operator sites alter rifampicin effects

To study the effects of rifampicin on gene expression, we first studied whether, at rifampicin concentrations that did not significantly affect cell growth rates, the effects were strong enough to be detectable at the protein level and differed according to promoter sequences.

Promoter sequences affect RNAP-promoter association and dissociation (28, 29, 65, 66), open complex formation (67), and RNAP-promoter escape (68, 69). Thus, they may influence the efficiency of rifampicin. However, asserting this using natural promoters is complex because they differ in many features and are influenced by distinct external factors. Thus, to test the influence of promoter sequences, we used a strain library (a kind gift from the Elf lab), where each strain carries a chromosome-integrated constitutive synthetic promoter (pFAB120) controlling the expression of the mVenusNB protein. Since the binding site is downstream from the TSS (Fig. 3A), LacI binding prevents RNAP from escaping the TSS. It is the sequence of this site that differs between strains by one to a few nucleotides, affecting LacI association and dissociation rates and, consequently, RNA production.

We half-induced the promoters (see “Bacterial strains”) and added rifampicin. We found that the relative changes in expression levels differed between strains (Fig. 3B; see File S2 at https://doi.org/10.5281/zenodo.21899680). *E. coli* could potentially make use of the promoter sequence dependence of the effects of rifampicin to alter its early response to rifampicin.

Similarly, the relative changes in single-cell variability in gene expression (calculated by the squared coefficient of variation, CV^2^) also differed (Fig. 3C). Most interestingly, a few strains differ in how much the mean changed while having the same changes in single-cell variability (e.g., O1M9 and O1M10). This suggests that the changes in mean and single-cell variability could be (partially) decoupled by rifampicin.

Overall, since promoter sequences are evolvable, these results also suggest the possibility that a few promoter sequences may have changed to tune gene-specific responses to rifampicin.

### Sublethal rifampicin stress triggers an adaptation process of the transcriptome

We further analyzed the transcriptome at 80 and 160 min after adding rifampicin (see “RNA-seq”; File S3). We first qualified each gene’s response relative to the control as: (i) activated if LFC > 1 (*P*-value < 0.05); (ii) repressed if LFC < −1 (*P*-value < 0.05); and (iii) non-responsive if −1 ≤ LFC ≤ 1 and/or *P*-value ≥ 0.05 (see “RNA-seq”).

**Fig 4:**
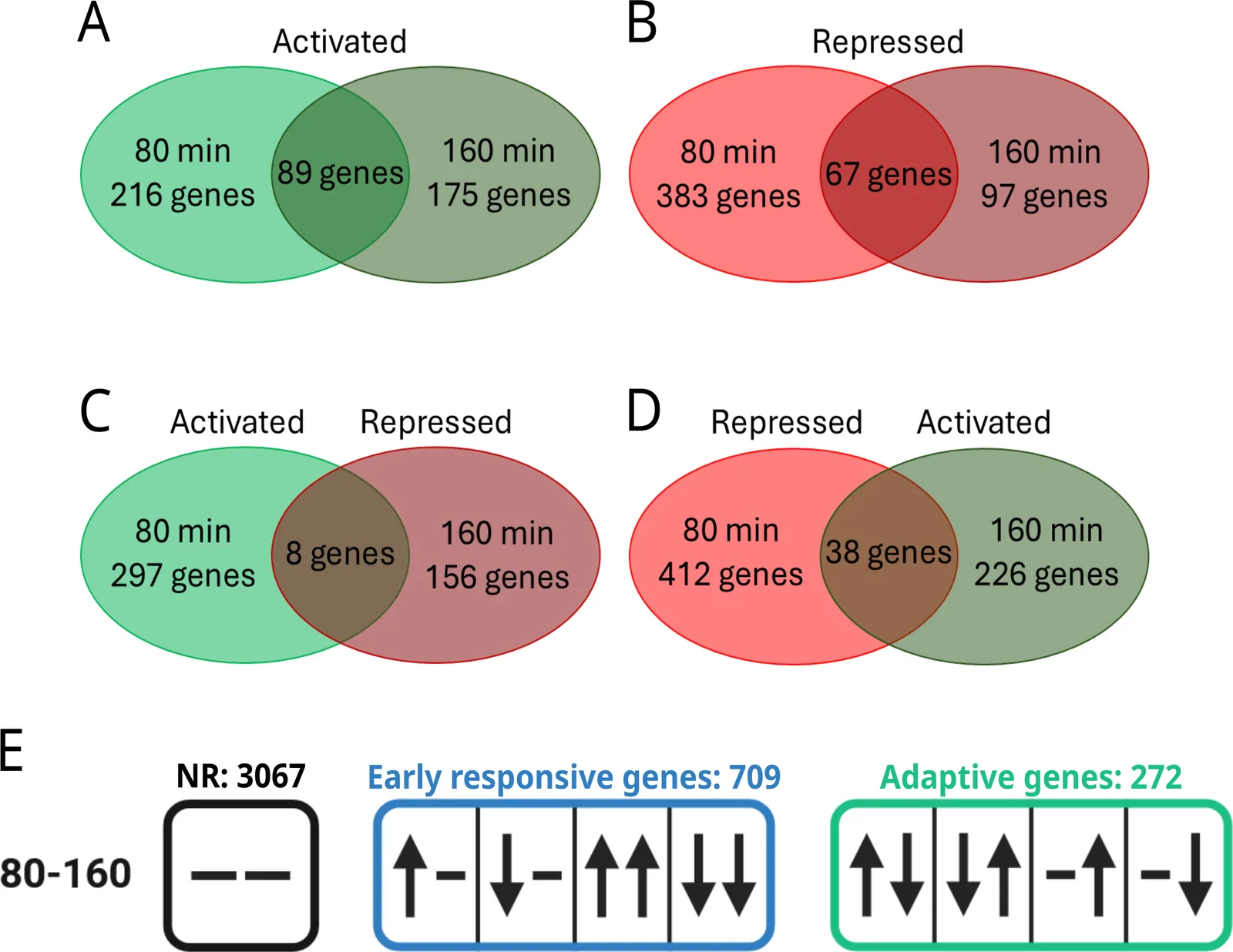
Early and late transcriptomic, GRN-driven responses to rifampicin. (**A**) Number of genes activated under rifampicin at 80, 160, or both 80 and 160 min. (**B**) Same as panel **A** but for repressed genes. (**C and D**) The numbers of genes activated at 80 but repressed at 160 min, and vice versa. (**E**) Number of genes with specific behaviors over time: activation/repression are represented by arrows pointing upward/downward, respectively. A minus sign means the behavior does not differ from the control. The blue and green boxes show the behaviors classified as “early responsive” and “adaptive,” respectively, while the black box is for non-responsive (NR) genes.

We found ~1,000 responsive genes (Fig. 4A through E). This agrees with rifampicin being a major distress and is in line with past work on transcription perturbations. For example, reductions of RNAP concentrations were found to affect a similar number of genes (51).

Among responsive genes, we found several temporal response patterns. Figure 4A through D shows the numbers of genes responsive at 80 and 160 min. At 80 min, genes were either activated or repressed relative to the control, indicated by an upward or downward arrow in the first position (Fig. 4E). By 160 min, some of these genes no longer differed from the control, indicated by a “−” in the second position. Other genes exhibited persistent responses (both arrows pointing in the same direction) or shifted their behavior (arrows pointing in opposite directions). This diversity of temporal patterns suggests that, while some behaviors are likely the direct effect of rifampicin, other behaviors may result from regulatory mechanisms.

To analyze these behaviors further, we next focused on the genes whose early behavior (at 80 min) differed from the control and did not “switch” later on (Fig. 4A and B). We identified 709 early responsive genes (Fig. 4E; File S4).

Next, we focused on genes that changed their behavior between 80 and 160 min, here identified as “adaptive” (Fig. 4C and D). This behavioral switching suggests that the mechanisms and/or effects regulating their short- and long-term behaviors are not the same. Thus, these are the most likely to drive the long-term adaptation, which usually involves later changes rather than just initial reactivity. Overall, we identified 272 “adaptive genes” (File S4). This relatively large number of genes suggests that the adaptation could involve significant transcriptional reprogramming.

Arguably, while some early responsive genes could also be actively contributing to the adaptation (albeit without switching behavior between short and long term), we excluded them to minimize false positives. Nevertheless, their number is surprisingly small, i.e., only ~20% of genes with early positive/negative responses remained positively/negatively responsive, respectively. All others either switched (~10%) or lost responsiveness in the long term.

Meanwhile, the decrease in the number of responsive genes from 80 to 160 min is in line with the early response, including genes directly affected by rifampicin rather than genes contributing to the adaptation alone. Contrarily, the late response was likely coordinated and restricted to genes contributing to the adaptation processes.

The conclusions above are based on RNA-seq data under rifampicin (2.5 µg/mL). At this concentration, cell growth rates are similar to control conditions (Fig. 2), while transcription is disrupted. Thus, overall RNA levels should become lower than in control cells (at least initially). This entails that some increases in RNA levels (particularly small ones) in the RNA-seq data could correspond to small decreases in absolute RNA levels. However, as illustrated in Fig. 1, we also expect changes due to the GRN mechanisms, such as TF.

For this to be possible, the protein levels of genes whose RNAs exhibit adaptive behaviors should also exhibit similar behaviors. To test this, we selected genes with adaptive behaviors (i.e., different behaviors at 80 and 160 min) whose RNA levels are higher than the control at 160 min alone (for easier detectability) and for which there are YFP strains available.

We found that the YFP strain library could track the activity of the genes *astC*, *bhsA*, *gltA*, *hisJ*, *phoH*, and *lldD*, classified above as adaptive. We thus measured their expression levels at 80 and 160 min with and without rifampicin (control condition). The results (LFC values relative to the control condition) are shown in Fig. S7, together with the corresponding RNA-seq measurements, for comparison.

From the figure, the expression levels of five out of the six genes behaved qualitatively similarly to the corresponding RNA-seq data. Shortly, aside from *GltA*, all gene expressions at 160 min were stronger than at 80 min. In detail, almost all levels were statistically significantly lower than the control at 80 min (except for AstC-YFP). In addition, all five levels increased significantly between 80 and 160 min.

The only condition that is not satisfied is that, contrary to the RNA-seq data, the levels at 160 min were not higher than the control, except for AstC and IldD. Potentially, this may be due to an increase in the time lag between YFP fluorescence levels relative to the corresponding RNA levels, caused by reduced translation and/or dilution rates of YFP as the cell population reaches the late exponential growth phase.

### Regulatory mechanisms controlling early responsive genes

#### Early behavior of global regulators, σ factors, and their downstream genes

We investigated the mechanisms controlling early responsive genes. We first considered global regulators (GRs) which, jointly, control a total of ~25% of all *E. coli* genes (55). Many are involved in stress responses. We investigated if they and their output genes show consistent, short-term responses under rifampicin. Since GRs activate some genes but repress others, we confronted their activities with the mean absolute LFCs of the output genes.

From Fig. S8 and Table S1, only *fnr*, which is required for switching to anaerobic respiration [55, 70]), and its output genes (71) have consistent and statistically significant responsiveness. Since *fnr*’s RNA numbers decreased, cells do not appear to be shifting toward an anaerobic program. This observation is in line with a recent study that reported that sustained aerobic respiration diminishes rifampicin killing efficacy (25).

We also tested if any GR could be responsible for the behavior of adaptive genes at 160 min, but we found no evidence (Table S2).

We also found that σ^70^ and σ^38^ levels were reduced significantly along with their target genes at 80 min (Table S3), but not at 160 min (Table S4). An early decrease in σ^70^ could be beneficial if it reduces commitment of the rifampicin-impaired RNAP pool to housekeeping σ^70^-promoters, shifting efforts from growth toward survival programs (72). Contrarily, an early decrease in σ^38^ probably is not beneficial, since this is a master regulator of stress responses. Thus, we interpret this decrease as a direct, non-beneficial effect of rifampicin. This is supported by the levels at the later stage, where they no longer differ from the control.

The list of σ-regulated genes is provided in File S5. Genes with dual σ factor regulation (less than 400 [55]) were included in the cohorts of both σ factors.

Overall, we conclude that a few GRs and output genes change consistently with a role in assisting the early, but not the late, programs of adaptation to rifampicin.

As a side note, we tested if any GR or σ factor could be responsible for the behavior of adaptive genes at 160 min, but only σ^32^ and its 11 output genes showed significant responsiveness (Tables S2 and S4). Since they are not known to contribute to long-term adaptations, this is likely a direct result of the disturbances caused by rifampicin rather than being related to the adaptation processes.

#### (p)ppGpp affects early responsive genes but not adaptive genes

**Fig 5:**
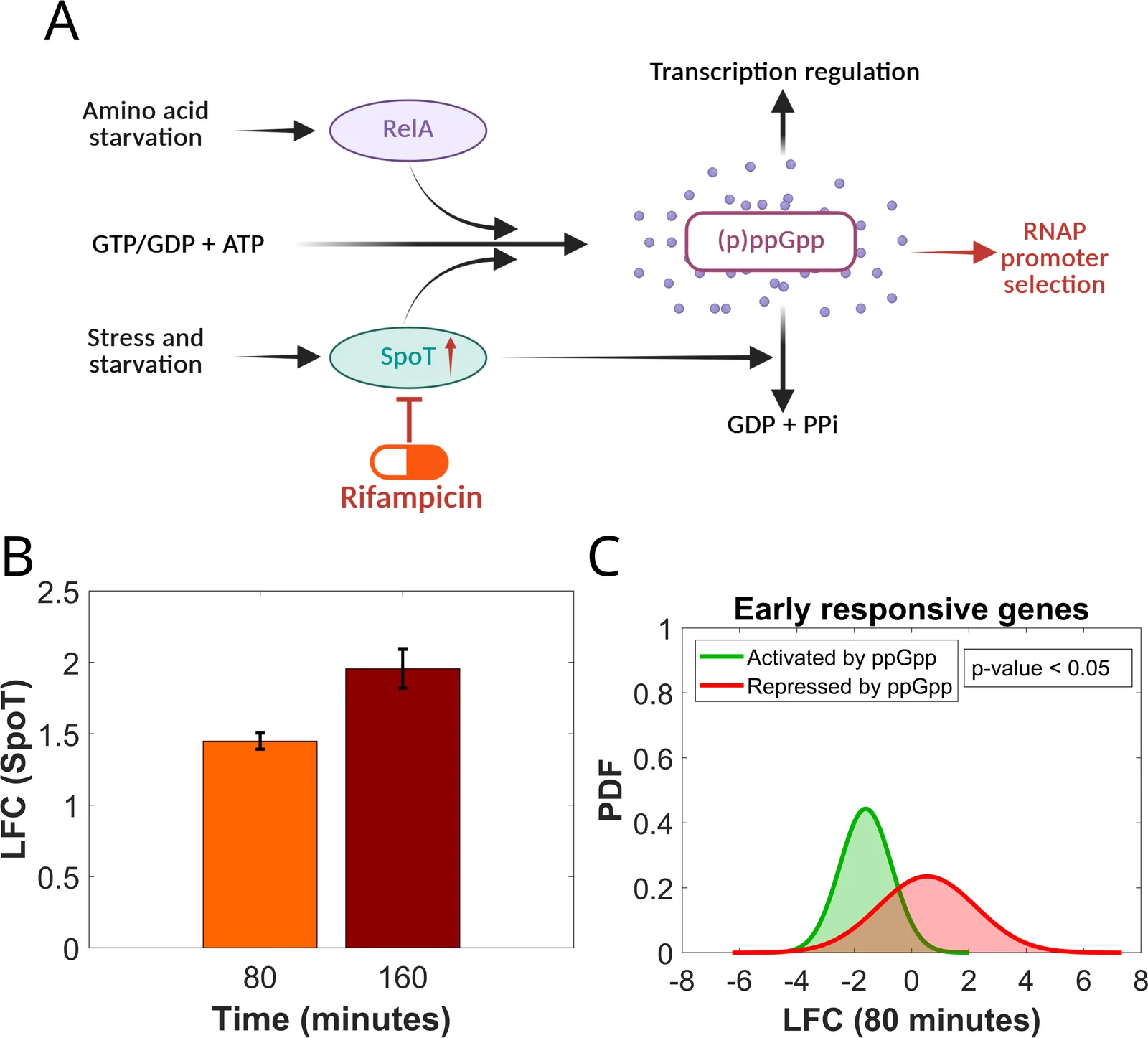
Influence of (p)ppGpp on the early responses to rifampicin. (**A**) Illustration of (p)ppGpp regulation controlled by *relA* and *spoT*. Adapted from reference 73. (**B**) SpoT-YFP levels relative to the control at 80 and 160 min. Error bars are the standard error of the mean from three biological replicates. (**C**) Probability density functions (PDF) of the LFC distributions at 80 min of early responsive genes known to be activated (µ = −0.97, SD = 0.96, and *N* = 182) and repressed by (p)ppGpp (µ = 0.10, SD = 0.85, and *N* = 99), respectively. Also shown is the *P*-value of a two-sample *t*-test to assess if the two distributions are from independent random samples from normal distributions with equal means and equal, but unknown, variances. For *P*-value <0.05, we reject the null hypothesis that the two distributions have equal means at the 5% significance level.

Rifampicin could affect the main regulators of (p)ppGpp, *spoT* and *relA* (20) (Fig. 5A). In agreement, we noted that the percentage of early responsive genes that are regulated by (p)ppGpp is higher than expected by chance (17% rather than 8%). We thus used a YFP strain to track spoT, which is responsible for the hydrolysis of (p)ppGpp in normal growth conditions.

From Fig. 5B, SpoT is higher than in the control condition in both the short and the long term. This should, in turn, decrease (p)ppGpp (74) and affect the 308 (p)ppGpp-regulated genes (RegulonDB v. 13.0 [55]). In agreement, most early responsive genes activated by (p)ppGpp were downregulated by rifampicin. Consistent with this, most early responsive genes repressed by (p)ppGpp were not downregulated (Fig. 5C; File S6).

Given this, some early transcriptome changes under rifampicin may be partially mediated by (p)ppGpp, rather than being only a direct consequence of transcription blocking. Contrarily, we found no evidence that (p)ppGpp affects the long-term behavior of adaptive genes.

#### Interactions between early responsive genes influence their dynamics

**Fig 6:**
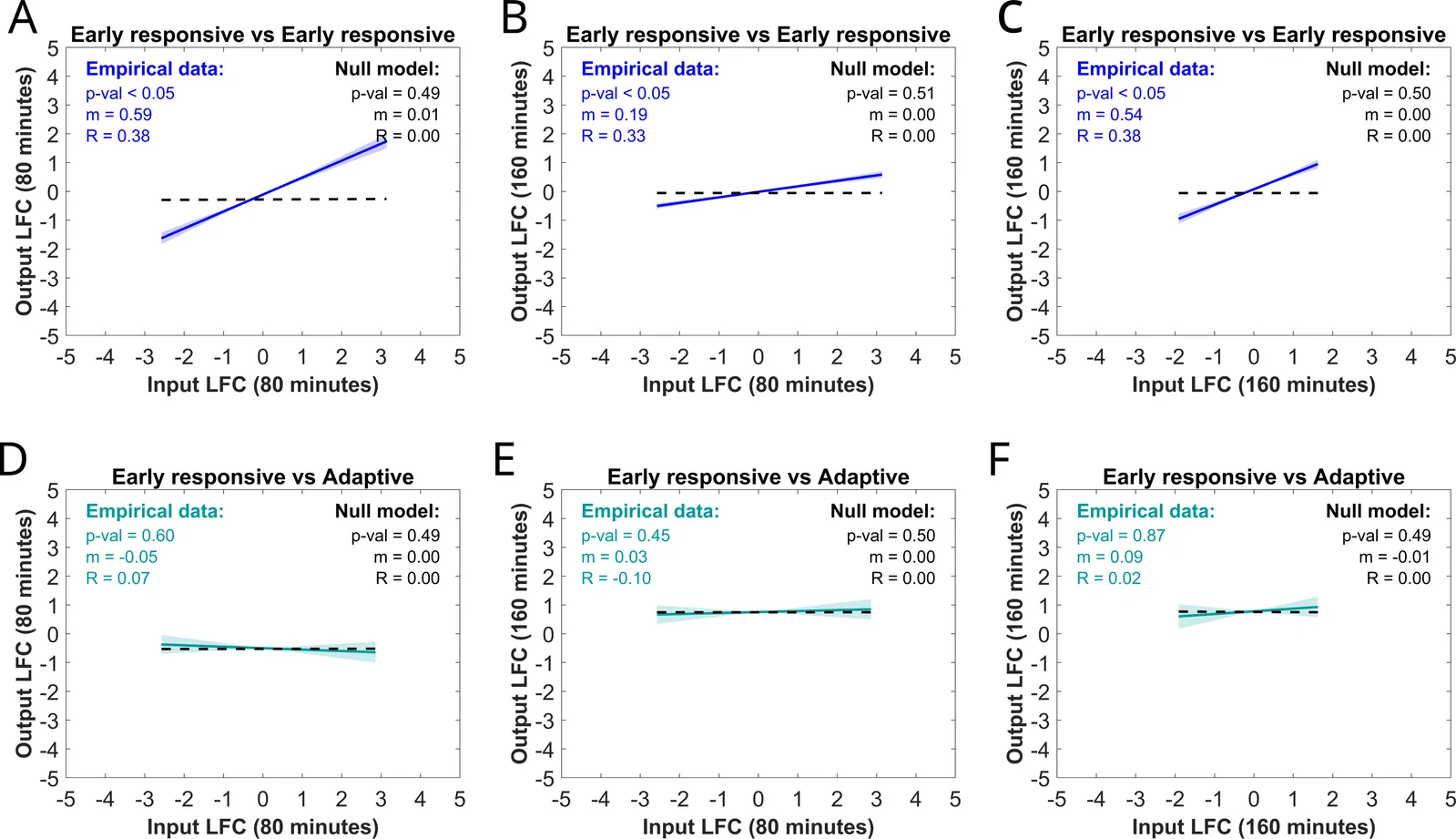
Interactions influence the early response to rifampicin. Correlation plots between the LFC of input and their output genes. (**A–C**) Correlation plots of the dynamics at 80 min for pairs of interactive early responsive genes, i.e., genes whose early behavior differed from the control and did not “switch” at 160 min. (**D–F**) Correlation plots of the dynamics of pairs of interactive genes (one early responsive at 80 min and one adaptive at 160 min). Adaptive genes are those whose behavior under rifampicin differed from the control at 160 min and also shifted the expression pattern between 80 and 160 min. The solid lines are the best fits, while shaded regions show the 68.2% confidence bands. *P* is the *P*-value for the regression slope. *R* is the Pearson correlation coefficient, and *m* is the slope of the line. For *P* < 0.05, we reject the null hypothesis that the slope is zero. Also shown are dashed lines, which are the linear fits to random models, where the interactions between genes are randomly switched between output genes (the line shown is the average fit from 1,000 random models). Auto-regulations are only considered in panels B and E, given that they evaluate correlations between 80 and 160 min.

We studied whether the behaviors of early responsive genes can, to some extent, result from regulation between them. Figure 6A through C shows the linear fit to scatter plots between the LFCs of early responsive genes known to interact.

Critically, in all three cases, the fitted lines differ statistically from horizontal lines, supporting the existence of correlation between input and output gene dynamics during the response to rifampicin. However, this correlation would also be observed if all genes were correlated for reasons other than interactions. To show that interactions are responsible for the correlations, we used a null model. Specifically, we randomized which genes are outputs of the interactions. When doing so, the correlations are no longer detectable (Fig. 6A through C). Overall, the results suggest that early responsive genes are not just co-responding to rifampicin; instead, interactions affect their responsiveness.

Interestingly, the *R*^2^ values in Fig. 6A through C are between 0.33 and 0.38. These can be considered high values, since the response strengths of output genes relative to input genes differ between gene pairs due to differing TF binding strengths and other factors, which disperses values from the fitted line. Interestingly, *R*^2^ = 0.38 for both Fig. 6A and C, suggesting similar behavioral correlations between input and output genes in the early and late stages of the stress. Meanwhile, the correlation is smaller (0.33) between 80 vs 160 min. This is in line with significant behavioral changes between the two time points.

Finally, we did not find correlated behaviors between input early responsive genes and output adaptive genes (Fig. 6D through F). This suggests that the adaptive GRN state is not a direct extension of the early state.

### Regulatory mechanisms controlling adaptive genes

#### Interactions between adaptive genes influence their long-term dynamics

As shown above, adaptive genes were not influenced by TFs and sRNAs controlled by other genes. Next, we studied whether TFs and sRNAs between adaptive genes were influential (see Files S4 and S7).

**Fig 7:**
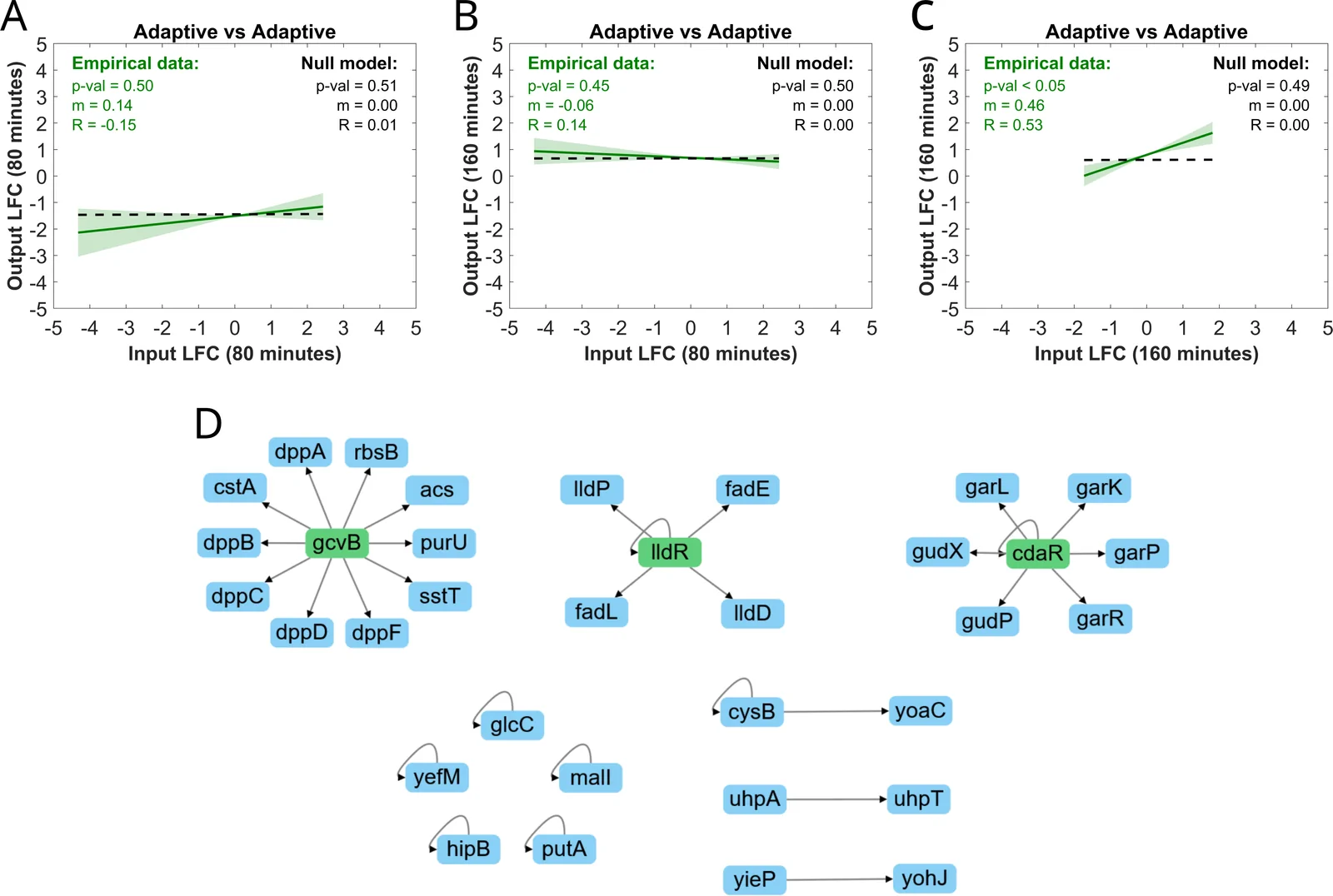
Interactions influence the late response of adaptive genes to rifampicin. (**A–C**) Correlation plots between the LFC of adaptive genes known to interact. The lines are the best linear fit. Shaded regions are the 68.2% confidence intervals. *P* is the *P*-value for the regression slope. For *P* < 0.05, we reject the null hypothesis that the slope is zero. Dashed lines result from a random model where the TF interactions are randomly switched between output genes (the final result is the average fit to 1,000 random models). Auto-regulations are only considered in panel B. (**D**) Visualization of the topology of the interactions between adaptive genes. Green nodes are the top three genes that regulate more adaptive genes. Network was designed using Cytoscape (75). *R* is the Pearson correlation coefficient, and *m* is the slope of the line.

Figure 7A through C shows the linear fit to a scatter plot of the LFCs of input adaptive genes vs the LFCs of output adaptive genes. We only observed a *P*-value < 0.05 when considering late responses, suggesting that the interactions influence the long-term behavior of adaptive genes to rifampicin (Fig. 7C). To validate, we again applied a null model. We found that there is no correlation if the interactions are between randomly selected adaptive genes (Fig. 7C). Finally, for short-term behaviors or between short- and long-term behaviors, *P*-values are much higher than 0.05, suggesting no influence (Fig. 7A and B).

These results suggest that long-term adaptation to rifampicin is likely partly coordinated by the interactions between adaptive genes. These interactions could be potential vulnerabilities of the program of adaptation to rifampicin.

#### Structure of the interactions between adaptive genes

Unlike individual genes, interactive genes can produce, e.g., feedback and switching behaviors, potentially allowing for stability, time counting, memory, etc. The capacity for such behaviors is expected to differ with the structure formed by the interactions between the genes. We studied the structure formed between adaptive genes, which should be responsible (at least partially) for the late dynamic correlations reported above. In detail, we searched for interactions reported in reference 55 between genes whose behavior under rifampicin differed from the control condition at 160 min and also shifted the expression pattern between 80 and 160 min (i.e., classified above as adaptive).

The structure of the interactions is shown in Fig. 7D. First, the size of this network of 34 genes is similar to the size of other networks known to drive adaptive programs, e.g., the SOS response involves ~30 genes (76), the phosphate starvation response involves ~31 genes (77), and the superoxide stress response involves ~15 genes (78).

In addition, from Fig. 7D, this network has three modules. The central regulators (hubs) of those modules are, respectively, *lIdR*, which is responsible for aerobic lactate utilization, *cdaR*, which codes for a DNA-binding transcriptional regulator that activates genes for uptake and catabolism of sugar diacids, and *gcvB*, which codes for an sRNA that post-transcriptionally represses several mRNAs (55).

All three modules have a star-like (high out-degree, centralized) structure, each with one hub that should be able to quickly coordinate all output genes. Since the modules are disconnected, they may operate relatively independently during the AB stress response. In contrast to its speed, its decision-making capacity is likely limited (i.e., it has only a limited number of transcriptional programs with small differences between them), fully determined by the state of the central genes.

In addition to the modules, there are eight self-regulated adaptive genes, three independent pairs of interacting adaptive genes, and no triangles. Out of the eight self-regulations, six are negative, one is positive (*cdaR*), and one can be positive as well as negative (*IldR*) (55). Such strong bias toward negative self-regulations suggests a preference for stability and fast responses, along with the dampening of cell-to-cell variability.

Overall, these results are consistent with reported cellular tuning of multiple biofunctions under stresses, followed by stabilization once an adaptive state is established.

### Orthologous genes of *E. coli* and *M. tuberculosis* response strengths to rifampicin are correlated

To test if our results are generalizable to other bacterial species, we compared with *M. tuberculosis* alone, since we did not find RNA-seq data under sublethal rifampicin concentrations at similar time points for other species. *M. tuberculosis* has been estimated to be carried by almost 2 billion people in recent years (79). Rifampicin is commonly used to treat tuberculosis in combination with other antimycobacterial drugs (80).

*M. tuberculosis* is evolutionarily distant from *E. coli* (different phyla that diverged long ago) (81). In addition, it has a higher GC content (~65%) (82) than *E. coli* (~50%) (83) and structurally different cell walls (84). The higher survivability of *E. coli* under rifampicin may be due to its lower membrane permeability along with more efficient membrane efflux pumps (85). Finally, physiologically, while *M. tuberculosis* is a pathogen that grows slowly and persists in host tissues (86), *E. coli* grows quickly and lives in the gut, usually without causing harm (87).

Data on *M. tuberculosis* (six strains) under sublethal rifampicin levels were obtained from the NCBI GEO database (GSE292409). It provides transcript abundance data in transcripts per million (TPMs). Using Mycobrowser (https://mycobrowser.epfl.ch/ as of 28 June 2024 [88]), we associated each locus and gene identifier with their corresponding TPM.

We first compared each gene’s log₂(TPM) between pairs of the six *M. tuberculosis* strains. The correlations were similar for all pairs and very high (Fig. S9; Table S5). Thus, their value can be used as a “maximum expected correlation” when comparing log₂(TPM) of different bacterial species.

Next, we compared these values with the TPMs of *E. coli* (see RNA-seq). For that, we searched for orthologous genes of *E. coli* and *M. tuberculosis* (i.e., genes evolved from a common ancestral gene) by retrieving the IDs of each *E. coli* gene and querying the NCBI Gene database (rentrez R package [89]) to identify orthologous genes in *M. tuberculosis*. We found 567 orthologous pairs (~12% of all genes; File S8). Comparing the log₂(TPM) of orthologous genes (at 80 and 60 min under rifampicin for *E. coli* and *M. tuberculosis*, respectively), we found a significant correlation (Fig. S10) (albeit weaker than between pairs of *M. tuberculosis* strains, as expected). This suggests that the adaptive strategy to rifampicin might be similar in many bacteria. Overall, this correlation also suggests that the biofunctions performed by the antibiotic-responsive genes might provide phenotypic advantages during sublethal rifampicin stress.

Finally, unfortunately, we failed to find data for comparing long-term responses and therefore could not determine whether orthologous genes have similar adaptive responses.

## DISCUSSION

Many AB-resistant strains are likely to have emerged from bacterial populations under sublethal levels of ABs (2, 6). Furthermore, it has been established that AB exposure can trigger large-scale transcriptional reprogramming, even at sublethal concentrations (90–92). Moreover, rifampicin-focused studies have shown that transcriptional regulation can modulate rifampicin tolerance in *M. tuberculosis* (63, 93).

Meanwhile, much is unresolved on how the GRNs of bacteria susceptible to transcription-targeting ABs can quickly coordinate the transition from early, large transcriptomic disturbances to late adaptive states, or how TF interactions (including between adaptive genes) contribute to those transitions. Nevertheless, indirect evidence is accumulating that this TF-driven coordination takes place. For example, Deter et al. (94) report that σ factors and TFs drive a coordinated transcriptional response under AB treatment.

We explored which regulatory mechanisms triggered the timed responsiveness of early and long-term adaptive genes to rifampicin, including a modular TF architecture associated with the long-term adaptive state. Our findings provide a mechanistic and dynamically organized model of *E. coli*’s transcriptional programs of rifampicin adaptation.

Our results suggest two distinct, short- and long-term transcriptomic states. Specifically, ~700 genes exhibited early response patterns that differed from the control. Meanwhile, 272 genes exhibited a long-term response pattern that differed from the control and from their own behavior in the short term. Of these 272 genes, only ~15% were short-term responsive while the remaining were, initially, unresponsive. Together, these numbers are consistent with a genome-wide adaptation program able to dampen the initial effects of rifampicin and transition the cell from transcriptional disorganization to a stable, long-term state, likely driving antibiotic adaptation.

During the early, transient state, although the rifampicin concentrations were low enough not to affect cell growth, there was a strong transcriptomic disturbance, with ~1,000 genes differing from the control. Had it not been rapidly dampened, such a disturbance could have compromised cell viability. This dampening was likely facilitated by increased RNAP concentrations, with global effects opposing those of rifampicin, together with changes in σ^70^, Fnr, and (p)ppGpp, which may have had more restricted effects. Potentially, targeting these regulators could disrupt the ability of *E. coli* to counter the initial effects of rifampicin. Finally, promoter sequences likely influenced rifampicin responsiveness, suggesting that promoter architecture may help tune the response of individual genes.

In addition to these mechanisms, interacting early responsive genes exhibited correlated responses in both the short and long term. Since these correlations are not detectable after randomizing the input–output relationships, they are unlikely to be due to a trivial global effect of rifampicin. Instead, TF-mediated interactions are affecting the dynamics of early responsive genes. Potentially, this may contribute to early cellular adaptations. Meanwhile, we found no correlation between TF-interacting early responsive genes and adaptive genes. This suggests that early responsive genes do not contribute directly to later adaptive changes.

Meanwhile, analysis of the late state of the GRN indicated that the network is influential in the late dynamics, with this correlation disappearing when the input–output relationships were randomized. Perhaps, this influence contributes to the emergence and/or maintenance of beneficial phenotypic adaptations. Because adaptive genes were not detectably influenced by TFs controlled by other genes, these results further suggest that the long-term state is coordinated mainly through interactions within the adaptive cohort itself. If these interactions are required to maintain the adaptive state, they may constitute vulnerabilities that could be targeted to disrupt adaptation to rifampicin.

The structure of the network of interactions between adaptive genes was also non-random. Rather than being dense or highly integrated, the interacting adaptive genes were largely organized into three disconnected, star-like modules centered on *lldR*, *cdaR*, and *gcvB*, together with a small number of self-regulated genes and isolated interacting pairs. This structure is consistent with long-term adaptation involving a limited number of relatively independent phenotypic adaptations. The bias toward negative self-regulation suggests the need for stabilization and reduced cell-to-cell variability once the adaptive state is established. Together, these observations support the possibility that adaptation to sublethal rifampicin is maintained by a small number of gene modules that may be themselves vulnerable to perturbations.

Furthermore, we studied whether the findings apply to an evolutionarily distant species, *M. tuberculosis*. We found that the response strengths of the orthologous genes correlate. This could be evidence for conserved rifampicin-responsive genes from a common ancestor, or that both species independently evolved similar phenotypic adaptations that are triggered by orthologs. Nevertheless, these observations suggest that new means to cope with AB adaptability developed for *E. coli* could be of use against other pathogens.

Several questions remain unanswered. For example, do TFs modify how rifampicin affects their output genes, and does this depend on their regulatory mode of action? Do some genes have more than one mechanism affecting their AB response? Arguably, genes underlying the most important adaptive responses may employ more than one response mechanism, which could allow for complex temporal control of their responsiveness. In this regard, it would be of interest to study the response to rifampicin of the network of deletion mutants for each of the three genes identified as hubs of the network of interactions among adaptive genes.

Finally, by dissecting the rifampicin-response mechanisms of natural genes, our findings could support the engineering of valuable synthetic circuits with scientific and/or biotechnological applications. For example, the ability of promoter sequences to affect how much rifampicin obstructs their RNA production rate could be of use to tune the responsiveness of future AB-responsive (dose-dependent) synthetic genetic circuits (e.g., genetic toggle switches, clocks, and signal filters). Similarly, the ability of TFs to propagate the effects of rifampicin could be used in the engineering of large rifampicin-responsive synthetic genetic circuits. Our findings could also have implications for the development of new ABs that, rather than targeting resistance, target the strategies of AB survivability applied by AB-susceptible species, which influences their ability to develop resistance.

## Data Availability

The RNA-seq data generated for this study are available in GEO under accession no. GSE304591. All other data are provided in the supplemental material. File S2 is available at https://doi.org/10.5281/zenodo.21899680.

## SUPPLEMENTAL MATERIAL

File S1 msystems.00808-26-s0001.xlsx Spectrophotometry data on the rifampicin effects on OD_600_, ATP, and gene expression.

File S3 msystems.00808-26-s0002.xlsx Gene name, LFC, and *P*-value at 80 and 160 min.

File S4 msystems.00808-26-s0003.xlsx Genes classified as "early responsive" and as "adaptive."

File S5 msystems.00808-26-s0004.xlsx σ-Factor-regulated genes.

File S6 msystems.00808-26-s0005.xlsx Positively and negatively (p)ppGpp-regulated genes.

File S7 msystems.00808-26-s0006.xlsx TF interactions reported in RegulonDB.

File S8 msystems.00808-26-s0007.xlsx Orthologous genes of *E. coli* and *M. tuberculosis* and their log_2_(TPM).

Legends msystems.00808-26-s0008.docx Legends for Files S1 to S8.

Supplemental material msystems.00808-26-s0009.pdf Supplemental figures and tables.

ASM does not own the copyrights to Supplemental Material that may be linked to, or accessed through, an article. The authors have granted ASM a non-exclusive, world-wide license to publish the Supplemental Material files. Please contact the corresponding author directly for reuse.
