# Supplementary material for: Distinct transcription factor network dynamics underlie early and long-term adaptation to rifampicin in Escherichia coli
Source: mSystems. 2026 Sep 2;11(9):e00808-26. doi: 10.1128/msystems.00808-26 (PMC13595921; doi:10.1128/msystems.00808-26)
Supplement: Supplementary file 1 [file msystems.00808-26-s0009.pdf]

Supplementary Information for

Distinct Transcription Factor Network Dynamics Underlie Early and Long-Term Adaptation to Rifampicin in Escherichia coli

M. Azevedo[1], A. Arsh[1,†], R. Jagadeesan[1,†], P. Ravaria[1], and A.S. Ribeiro[1,*]

[1] Laboratory of Biosystem Dynamics, Faculty of Medicine and Health Technology, Tampere University, Tampere, 33520, Finland.

[†] These authors contributed equally to this work

* To whom correspondence should be addressed. E-mail: andre.sanchesribeiro@tuni.fi.

Present Address: Andre S. Ribeiro, Arvo Ylpön katu 34, 33520, Tampere University, Finland.

Supplementary Figures

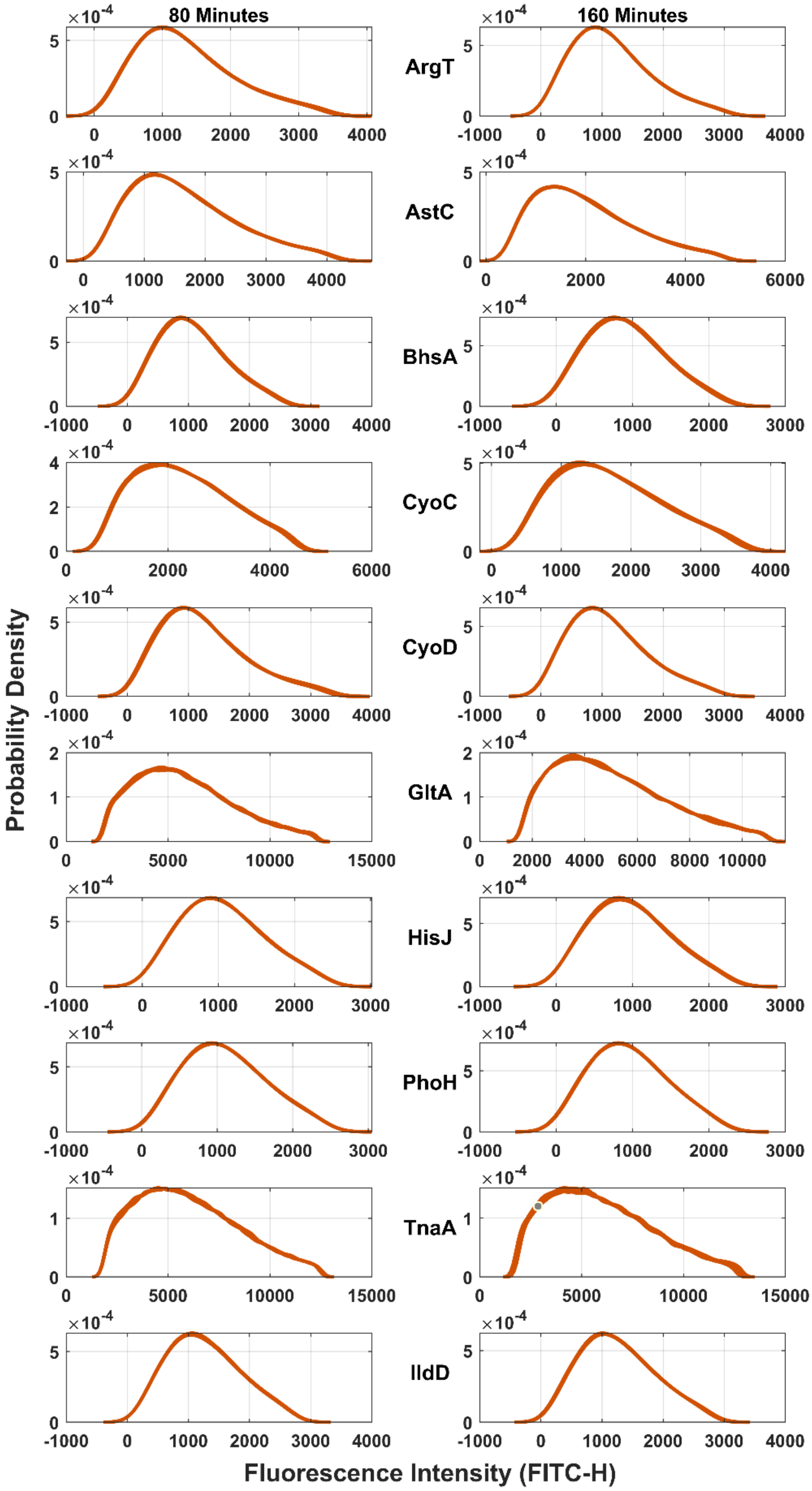

Supplementary Figure S1. Probability density functions of the distributions of single cell fluorescence intensities of cells from the YFP strain library tagging promoters of interest

(named between each pair of plots). Measurements at 80 minutes (left) and at 160 minutes (right) after adding 2.5 μg/mL of rifampicin.

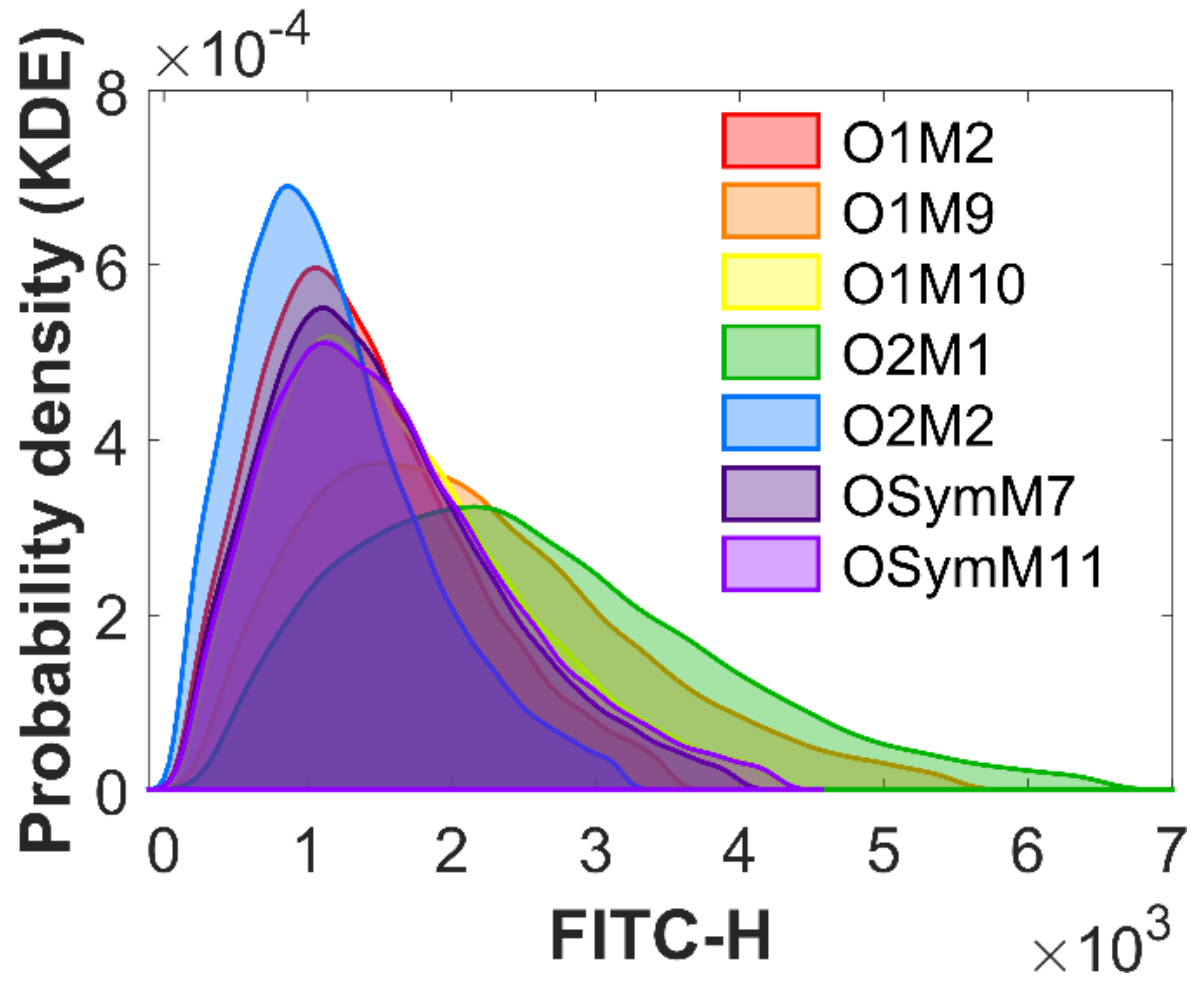

Supplementary Figure S2. Empirical single-cell distributions of protein levels as measured by flow-cytometry. Kernel density estimates (KDE) of the single-cell distributions of mVenusNB protein expression levels under the control of apFAB120 promoters with downstream LacI binding sites with unique single nucleotide mutations (named O1M2, O1M9, O1M10, O2M1, O2M2, OSymM7, and OSymM11), 40 minutes after adding 2.5 μg/mL of rifampicin. Negative values and the bottom and top 2% of values were excluded.

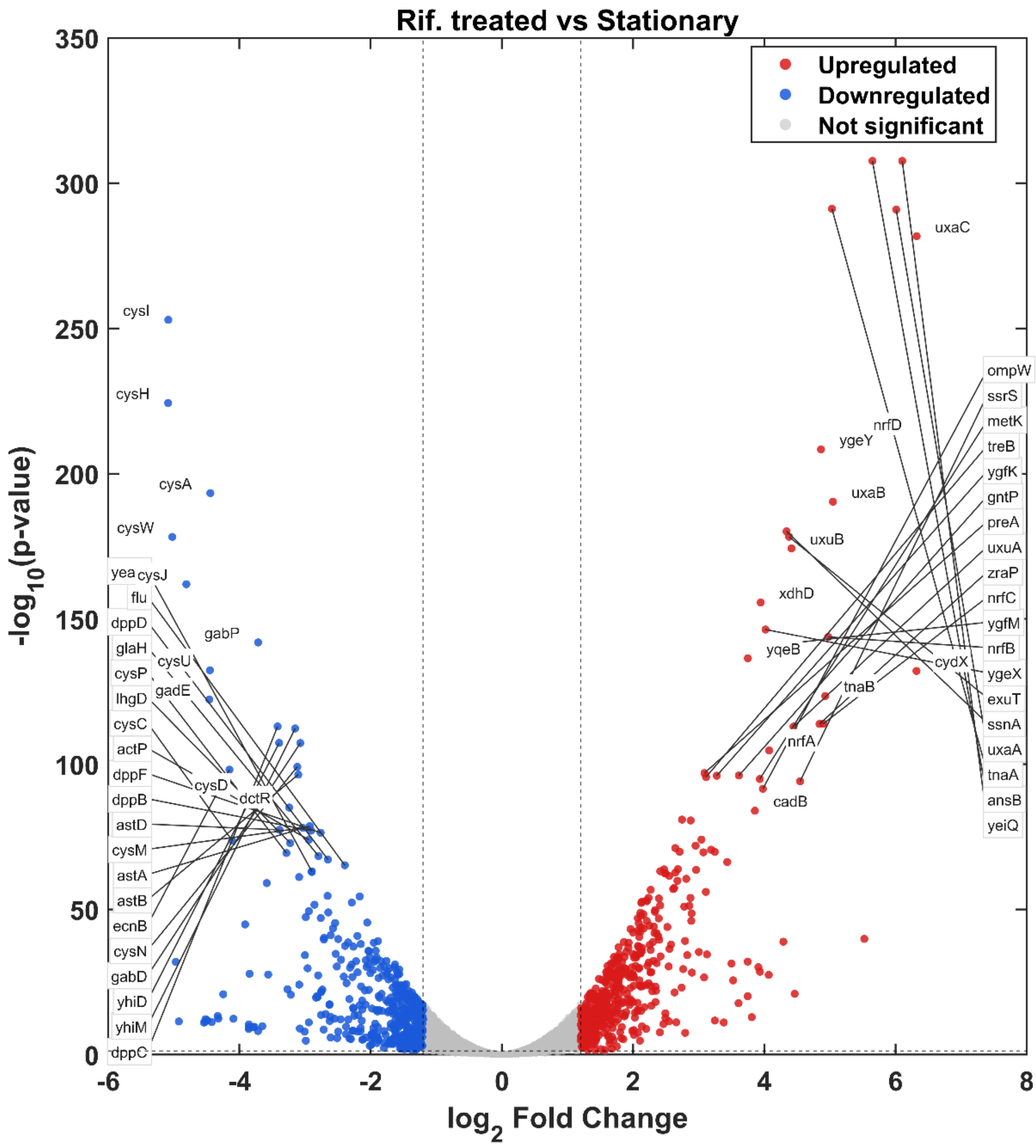

Supplementary Figure S3. Volcano plots of rifampicin treated cells (after 160 min.) versus stationary-phase cells. Shown is the distribution of Log_2 fold changes between rifampicin-treated cells at 160 min and cells in stationary growth phase. The x-axis represents the log_2 fold change in transcript abundance, while the y-axis represents the statistical significance as $-log_{10}$(adjusted p-value). Upregulated genes are shown in red, downregulated genes are in blue, and non-significantly regulated genes are in grey.

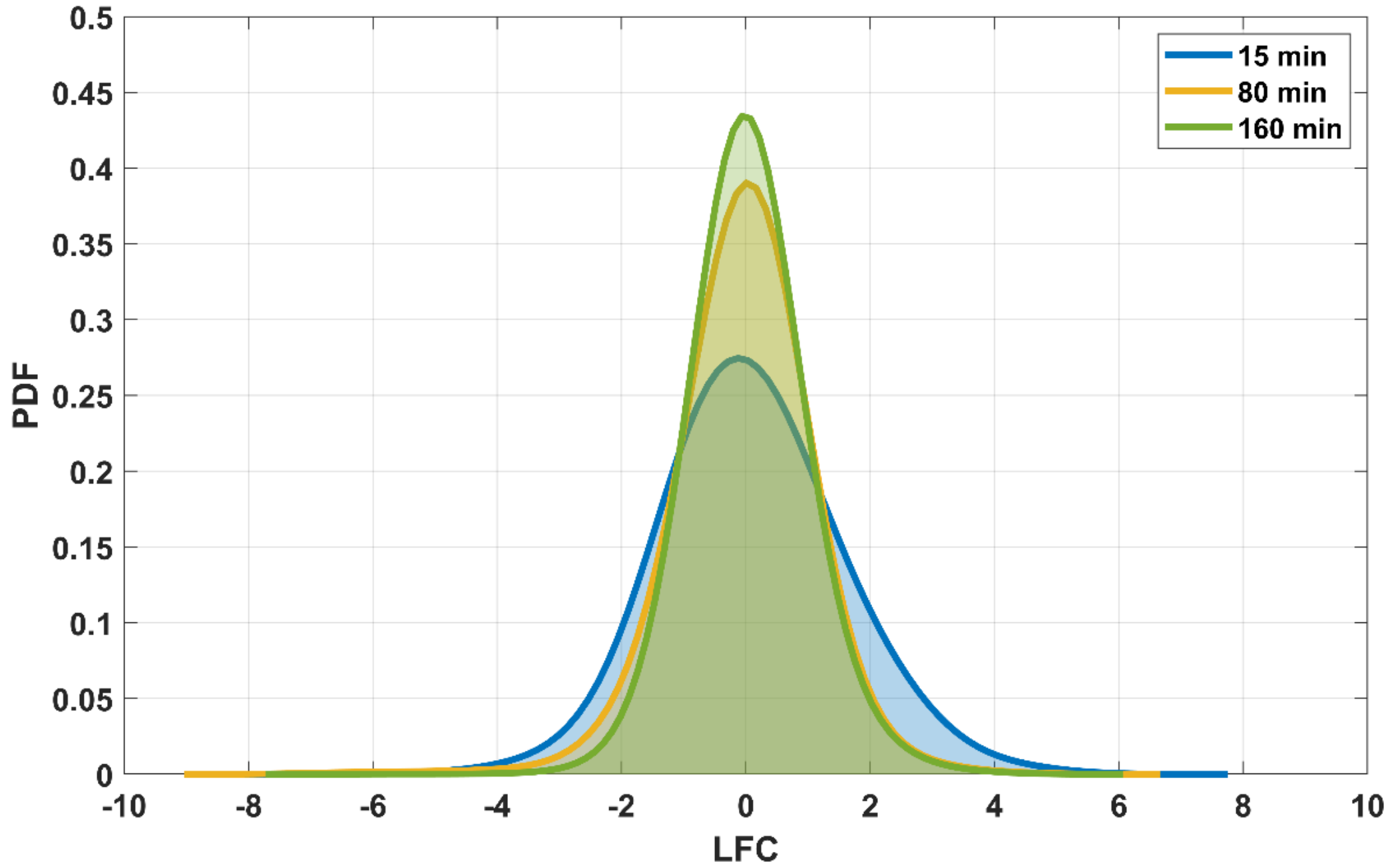

Supplementary Figure S4. Genome-wide probability density functions (PDF) of the distributions of LFCs at 15 minutes, 80 minutes, and 160 minutes after adding rifampicin, as measured by RNA-seq. The mean and standard deviations (μ, std) of these distributions equal (0.09, 1.4), (0.04, 0.94), and (0.04, 0.70) while the number of genes composing them are 3842, 4047, and 4067, respectively.

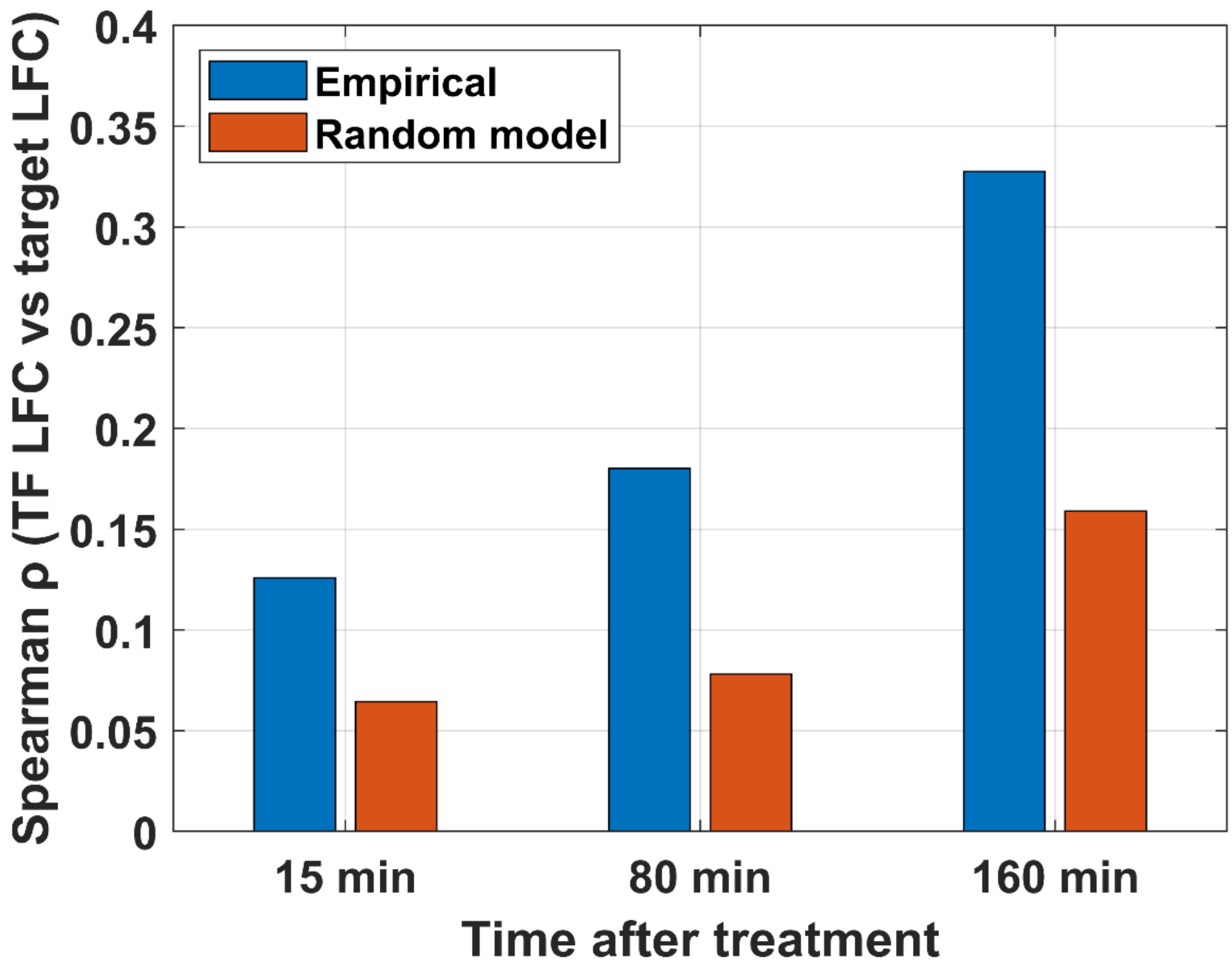

Supplementary Figure S5: Average correlation between the LFCs of transcription factor (TF) input-output genes after 15, 80, 160 minutes under rifampicin (Blue bars). Also shown is the maximum correlation obtained from a null model that randomizes which genes are outputs of the TF interactions (1000 runs).

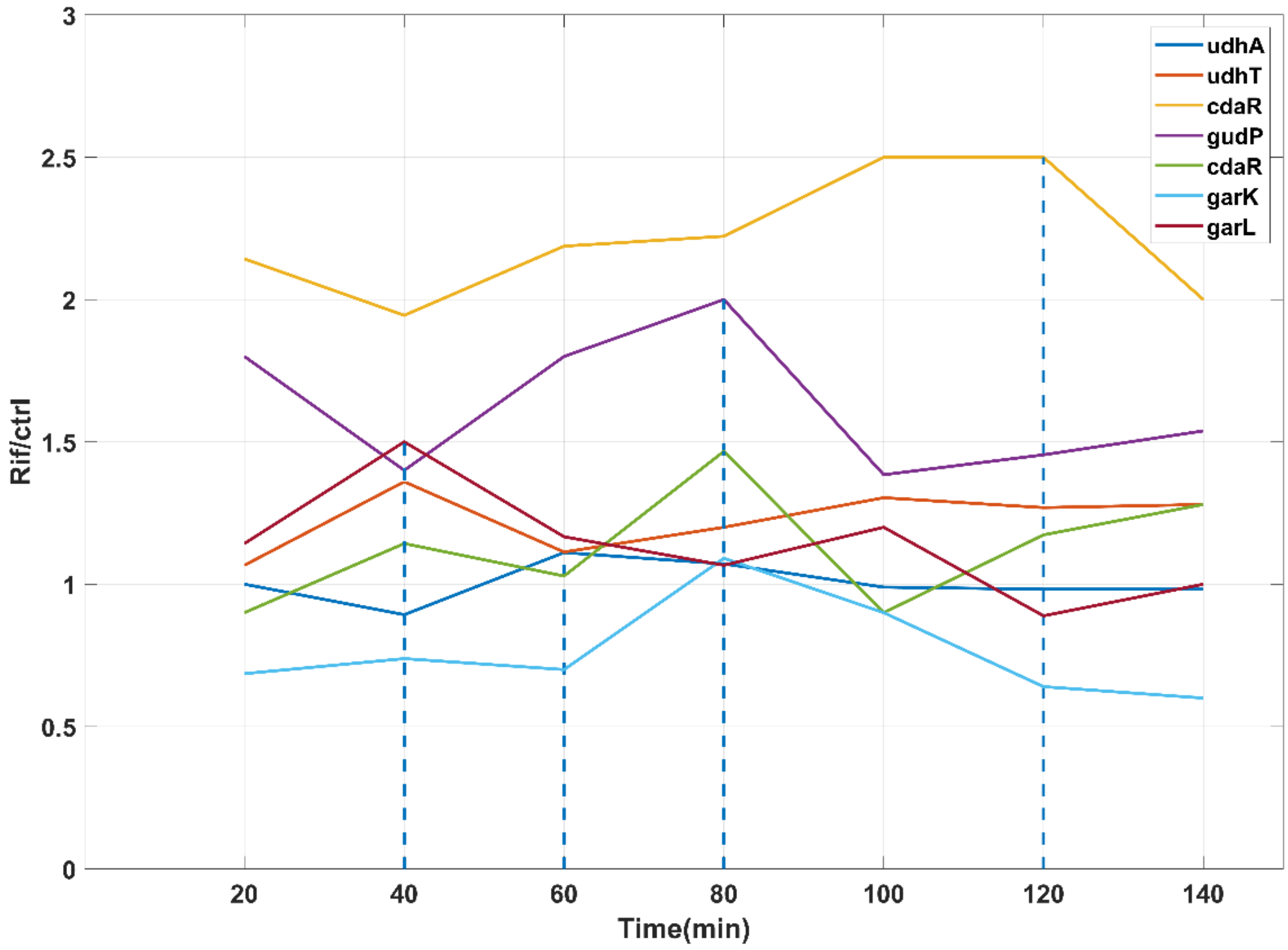

Supplementary Figure S6. Expression levels measured by spectrophotometry over time under rifampicin (Rif) relative to the control (ctrl) condition. Data obtained using strains from the YFP strain library (25).

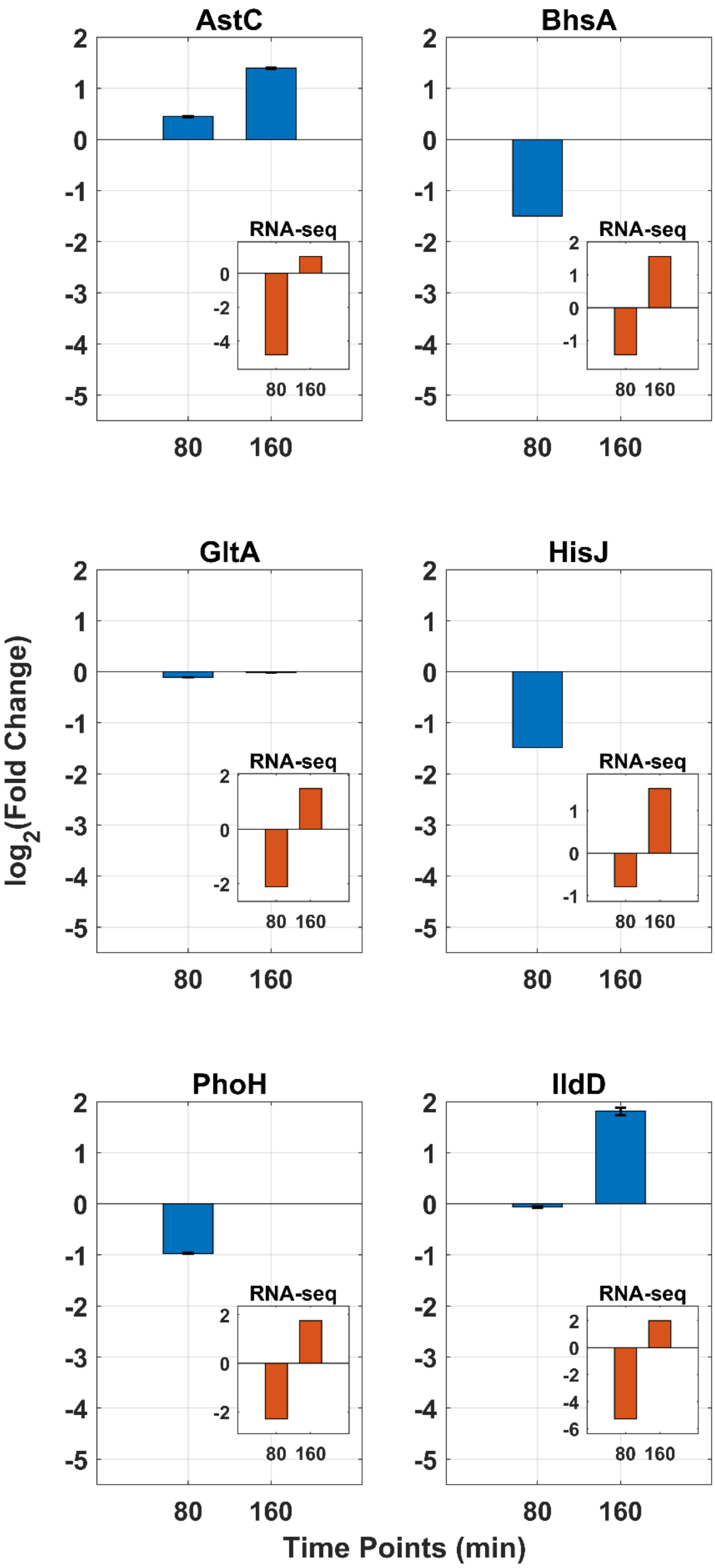

Supplementary Figure S7. Bar plot of the difference in average single-cell expression levels relative to the control condition at 80 and at 160 minutes after adding rifampicin, as measured

by flow-cytometry (blue bars). Shown are seven genes with, according to the RNA-seq data, exhibited adaptive behaviors such that only at 160 minutes had expression levels higher than the control. Error bars are the standard error of the mean (SEM) from 3 biological replicates. Also shown for comparison are the corresponding RNA-seq data for each gene (orange bars).

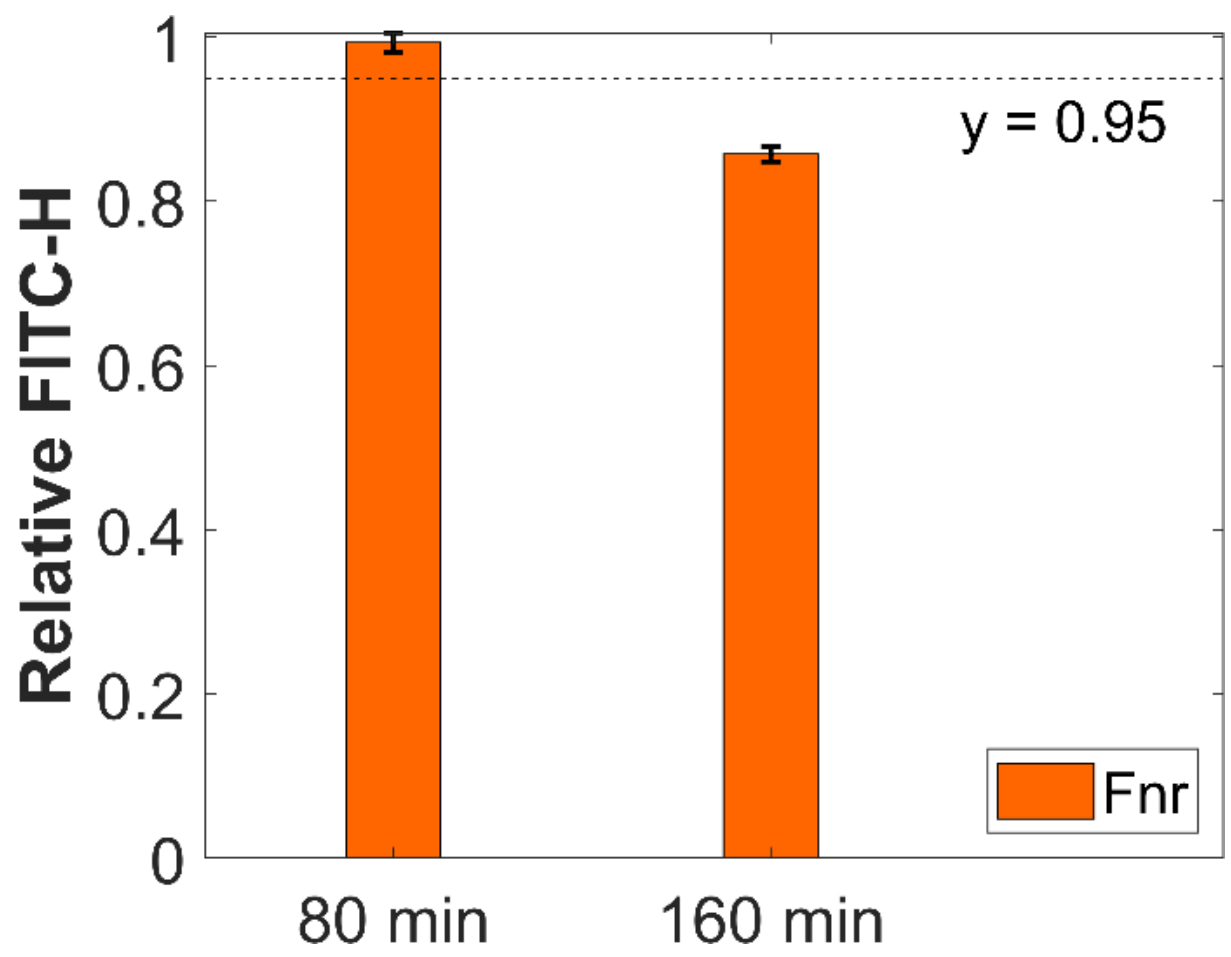

Supplementary Figure S8: Mean single-cell expression levels relative to the control of a GR, Fnr, at 80 and 160 minutes after adding rifampicin (measured by flow-cytometry). Error bars are the standard error of the mean (SEM) from 3 biological replicates. The dashed horizontal line at y = 0.95 marks a 5% difference from 1, below which we conclude that it differs significantly from 1 and, thus, that it differs from the control.

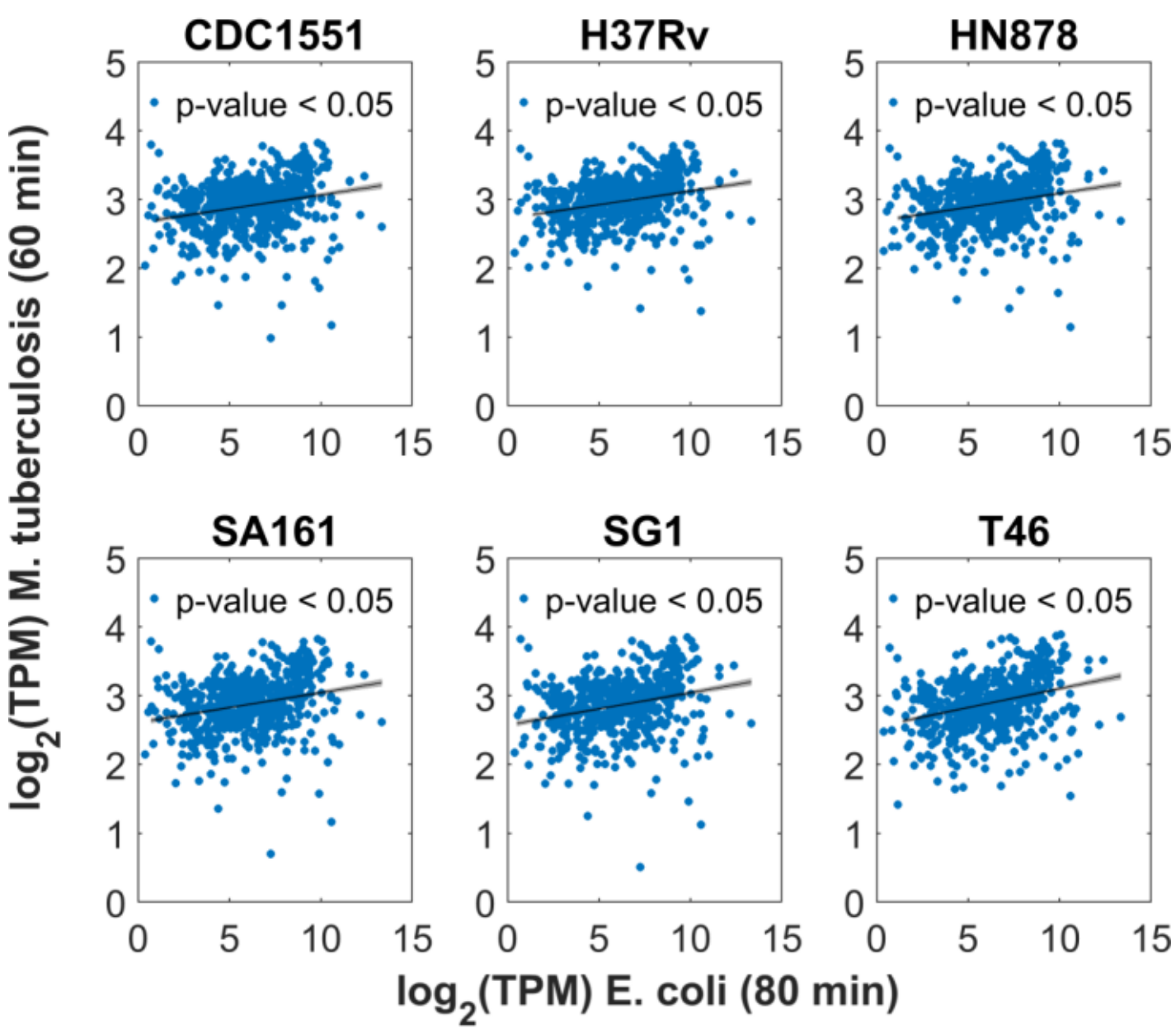

Supplementary Figure S9: Scatter plots of the TPMs of orthologous genes of *E. coli* and various *M. tuberculosis* strains (named on top of each graph) after 80 and 60 minutes under non-lethal concentration of rifampicin, respectively. Shown also are best fitting lines, the 95% confidence bounds (very small shadow areas), and the p-value under the null hypothesis that the line is horizontal. The hypothesis is not rejected for p value < 0.05. Supplementary Table S5 shows the values of m and R^2 of each best fit.

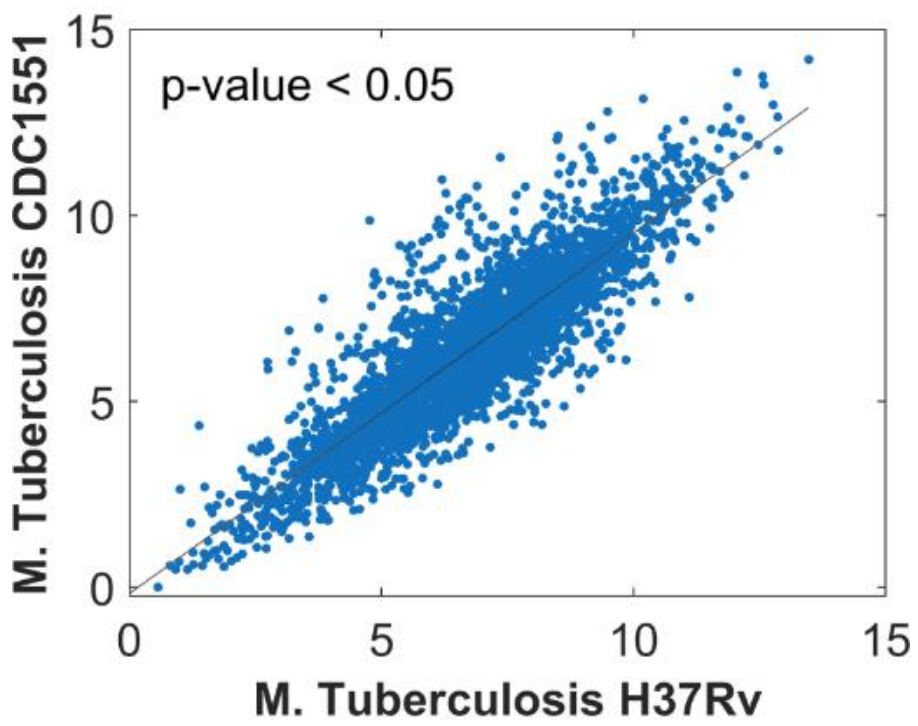

Supplementary Figure S10: Scatter plot of the TPM of the same genes from two strains of *M. tuberculosis* (H37Rv and CDC1551) 60 minutes under a non-lethal concentration of rifampicin. Shown also is the best fitting line with 95% confidence bounds (very small shadow areas) and the p-value under the null hypothesis that the line is horizontal. The hypothesis is not rejected for p < 0.05. The line inclination m equals 0.97 and the coefficient of determination R^2 equals 0.77.

Supplementary Tables

Supplementary Table S1: Mean μ and standard deviation STD of the LFC of the output, short-term responsive genes under the control of GRs, at 80 minutes. N is the number of short-term responsive genes controlled by the GR and p_1 is the probability that the distribution is the same as the distribution of all other genes (ttest2 MATLAB function). Also shown are the changes in RNA levels (LFC) of the GRs with rifampicin, at 80 minutes, followed by p_2, the p-value of their LFC. Only *fnr* and its output genes exhibit consistent behaviours, while differing from the control (marked with *).

**Regulated genes**	**μ(|LFC|)**	**STD (|LFC|)**	**N**	p_1	**GR**	**GR (|LFC|)**	p_2
crp	2.01	1.01	160	<0.05	*crp*	0.06	0.62
*fnr**	2.00	0.97	88	<0.05	*fnr**	(-) 0.40	<0.05
fis	1.67	0.57	48	0.90	*fis*	1.28	<0.05
narL	1.49	0.50	37	0.22	*narL*	0.01	0.93
fur	1.70	0.52	72	0.61	*fur*	0.02	0.88
lrp	1.66	0.76	80	0.95	*lrp*	0.03	0.81
nsrR	1.58	0.25	14	0.74	*nsrR*	0.08	0.62
cra	1.78	0.85	23	0.47	*cra*	0.71	<0.05
flhD	1.27	0.23	8	0.19	*flhD*	0.92	<0.05
cpxR	1.57	0.49	16	0.68	*cpxR*	0.41	<0.05
narP	1.40	0.23	17	0.20	*narP*	0.29	0.05
phoB	1.60	0.61	13	0.81	*phoB*	0.12	0.49
lexA	1.71	0	1	0.94	*lexA*	0.13	0.27

phoP	1.56	0.41	12	0.69	*phoP*	0.30	<0.05
marA	2.33	1.54	14	<0.05	*marA*	0.09	0.74
soxS	1.51	0.43	9	0.61	*soxS*	0.05	0.84

Supplementary Table S2: Mean μ and standard deviation STD of the LFC of the output, adaptive genes under the control of GRs, at 160 minutes. N is the number of adaptive genes controlled by the GR and p_1 is the probability that the distribution is the same as the distribution of all other genes (ttest2 MATLAB function). Also shown are the changes in RNA levels (LFC) of the GRs with rifampicin, at 160 minutes, followed by p_2, the p-value of their LFC.

Regulated genes	μ (|LFC|)	STD (|LFC|)	N	p_1	GR	GR (|LFC|)	p_2
crp	1.52	0.41	54	0.89	*crp*	0.37	0.11
fnr	1.62	0.37	36	0.33	*fnr*	0.01 (-0.01)	0.94
fis	1.57	0.37	29	0.71	*fis*	0.29	0.29
narL	1.48	0.35	6	0.84	*narL*	0.43	0.07
fur	1.59	0.36	34	0.50	*fur*	0.22	0.34
lrp	1.38	0.26	33	0.14	*lrp*	0.33	0.10
nsrR	1.46	0.38	6	0.78	*nsrR*	0.08	0.77
cra	1.59	0.38	9	0.76	*cra*	0.52	<0.05
flhD	1.50	0.34	12	0.88	*flhD*	0.53	0.28
cpxR	1.67	0.69	7	0.52	*cpxR*	0.14	0.49
narP	1.48	0.35	6	0.84	*narP*	0.09	0.69

phoB	1.55	0.27	5	0.93	*phoB*	0.22	0.36
lexA	1.36	0.34	4	0.55	*lexA*	0.76	<0.05
phoP	1.36	0.29	3	0.61	*phoP*	0.38	0.07
marA	1.41	0.24	4	0.38	*marA*	0.72	<0.05
soxS	1.46	0.14	3	0.84	*soxS*	0.11	0.77

Supplementary Table S3: Differences in RNA levels from the control of genes coding for σ factors as well as of the cohort of genes that they regulate, at 80 minutes after adding rifampicin. μ and SD are the mean and standard deviation of LFC values of σ-regulated genes, respectively. N is the number of those genes. Finally, p_1 is the probability that the distribution is the same as the distribution of all non-σ regulated genes and is given by the ttest2 MATLAB function, while p_2 is the p-value of the LFC value. Only σ^{38} and σ^{70} and their regulated genes, respectively, exhibit consistent behaviours, while differing from the control (marked with *).

**Regulated genes**	**μ(LFC)**	**SD(LFC)**	**N**	**p_1**	**σ factor**	**LFC**	**p_2**
No σ regulated genes	-0.06	1.98	305	<0.05	-	-	-
σ^{19} regulated genes	-	-	0	-	σ^{19}	0.78	0.19
σ^{24} regulated genes	-0.40	1.83	8	0.86	σ^{24}	-1.59	<0.05
σ^{28} regulated genes	-1.80	1.83	12	<0.05	σ^{28}	0.01	0.99
σ^{32} regulated genes	-0.55	1.84	11	0.64	σ^{32}	-0.75	<0.05
σ^{38} regulated genes*	-1.82	1.69	126	<0.05	σ^{38}	-0.31	<0.05
σ^{54} regulated genes	-1.48	1.77	38	<0.05	σ^{54}	-0.10	0.36
σ^{70} regulated genes*	-0.10	1.70	301	<0.05	σ^{70}	-0.19	<0.05

Supplementary Table S4: Differences in RNA levels from the control of genes coding for σ factors as well as of the cohort of genes that they regulate, 160 minutes after adding rifampicin. μ and SD are the mean and standard deviation of LFC values of σ-regulated genes, respectively. N is the number of those genes. Finally, p_1 is the probability that the distribution is the same as the distribution of all non-σ regulated genes and is given by the ttest2 MATLAB function, while p_2 is the p-value of the LFC value. Only σ^{32} and σ^{32} regulated genes exhibit consistent behaviour, differing from the control (marked with *).

**Regulated genes**	**μ(LFC)**	**SD(LFC)**	**N**	**p_1**	**σ factor**	**LFC**	**p_2**
No σ factor regulated genes	0.41	1.48	113	0.37	-	-	-
σ^{19} regulated genes	-	-	0	-	σ^{19}	0.98	<0.05
σ^{24} regulated genes	1.16	1.56	6	0.30	σ^{24}	-1.03	<0.05
σ^{28} regulated genes	-	-	0	-	σ^{28}	0.44	0.51
σ^{32} regulated genes*	-1.36	1.51	19	<0.05	σ^{32}	-0.53	<0.05
σ^{38} regulated genes	1.01	1.57	20	0.13	σ^{38}	-0.25	0.23
σ^{54} regulated genes	1.17	1.55	19	0.06	σ^{54}	0.22	0.38
σ^{70} regulated genes	0.64	1.62	134	0.19	σ^{70}	0.10	0.64

Supplementary Table S5: Parameter values m, p value, and R^2 of the fitting lines to the correlation plots between the TPMs of orthologous genes of *E. coli* and *M. tuberculosis* after 80 and 60 minutes under non-lethal concentration of rifampicin, respectively.

**Strain**	**m**	**p value**	**R^2**
CDC1551	0.04	< 0.05	0.04
H37Rv	0.04	< 0.05	0.06

HN878	0.04	< 0.05	0.05
SA161	0.04	< 0.05	0.05
SG1	0.05	< 0.05	0.05
T46	0.05	< 0.05	0.08
