# Supplementary material for: Distinct transcription factor network dynamics underlie early and long-term adaptation to rifampicin in Escherichia coli
Source: mSystems. 2026 Sep 2;11(9):e00808-26. doi: 10.1128/msystems.00808-26 (PMC13595921; doi:10.1128/msystems.00808-26)
Supplement: Supplementary file 2 [file msystems.00808-26-s0008.docx]

**SUPPLEMENTARY DATA**

**Supplementary Information**

**Supplementary File S1:** Spectrophotometry data on the rifampicin effects on OD_600_, ATP, and gene expression.

**Supplementary File S2:** Flow-cytometry data on rifampicin effects on RpoB, RpoC, chromosome-integrated Lac promoter mutants, *spoT*, *cdaR*, *garL*, *garK*, *gudP*, *udhA*, and *udhT*, along with several genes tagged with YFP. Available at <https://doi.org/10.5281/zenodo.21899680>.

**Supplementary File S3:** Gene name, LFC and p-value at 80 and 160 minutes.

**Supplementary File S4**: Genes classified as “early responsive” and as “adaptive”, respectively.

**Supplementary File S5:** List of σ-factor regulated genes.

**Supplementary File S6:** List of positively and negatively (p)ppGpp-regulated genes.

**Supplementary File S7:** List of TF interactions reported in RegulonDB (12/02/2025).

**Supplementary File S8:** List of orthologous genes of *E. coli* and *M. tuberculosis* and their log₂(TPM).
